# NLK facilitates Caspase‐8 activation to drive macrophage PANoptosis in sepsis

**DOI:** 10.1002/ctm2.70616

**Published:** 2026-02-11

**Authors:** Yun Xia, Ren‐qi Yao, Hui‐fan Liu, Guo‐qing Jing, Delida Aidebaike, Jing Zuo, Shi‐qi Wan, Die Wu, Peng‐yue Zhao, Hai‐long Gong, Xing Wang, Hui‐min Zhou, Ning Dong, Yao Wu, Shi Liu, Xiao‐jing Wu, Yong‐ming Yao, Xue‐min Song

**Affiliations:** ^1^ Department of Anesthesiology Zhongnan Hospital of Wuhan University Wuhan China; ^2^ Department of General Surgery the First Medical Center of Chinese PLA General Hospital Beijing China; ^3^ State Key Laboratory of Virology Modern Virology Research Center College of Life Sciences Wuhan University Wuhan China; ^4^ Translational Medicine Research Center Medical Innovation Research Division and the Fourth Medical Center of Chinese PLA General Hospital Beijing China; ^5^ Department of Anesthesiology People's Hospital of Wuhan University Wuhan China

**Keywords:** Caspase‐8, inflammatory cell death, macrophage, Nemo‐like Kinase, PANoptosis, sepsis

## Abstract

Mounting evidence indicates that macrophage PANoptosis—an integrated inflammatory cell‐death program comprising pyroptosis, apoptosis, and necroptosis—plays a pivotal role in sepsis pathogenesis. However, its upstream regulation remains poorly understood. Here, we identify Nemo‐like kinase (NLK) as a novel regulator of Caspase‐8–mediated PANoptotic signalling in sepsis. Integrated analyses of bulk (GSE65682) and single‐cell (GSE167363) transcriptomic datasets from patients with sepsis revealed elevated NLK expression in monocytes, strongly associated with PANoptotic effectors and adverse outcomes. Functional studies using NLK conditional knockout mice driven by Csf1r‐iCre and lipopolysaccharide‐stimulated bone‐marrow‐derived macrophages showed that NLK deficiency attenuated Caspase‐8 cleavage, suppressed pyroptotic (cleaved Caspase‐1, GSDMD‐N) and apoptotic (cleaved Caspase‐3/7) activation, and redirected cell‐death execution towards a necroptosis‐dominant program (p‐RIPK1/3, p‐MLKL). This death‐mode redistribution was associated with attenuated cytokine release, reduced multiorgan injury, and improved survival. Mechanistically, NLK associated with the N‐terminal death effector domains (DEDs; amino acids 1–216) of Caspase‐8, thereby enhancing the efficiency of Caspase‐8 recruitment and proximity‐induced activation within FADD–RIPK1/3‐containing PANoptosome complexes. NLK deletion impaired Caspase‐8 activation within these complexes and promoted RIPK1–RIPK3 necrosome assembly. Moreover, Caspase‐8 overexpression in NKO macrophages partially restored GSDMD and Caspase‐3 cleavage and reduced p‐MLKL, confirming that NLK is required for efficient Caspase‐8 activation and optimal PANoptotic signalling. Collectively, these findings identify NLK as a regulatory rheostat of Caspase‐8–associated PANoptosis in sepsis and highlight the NLK–Caspase‐8 axis as a potential therapeutic target for fine‐tuning sepsis‐associated inflammatory cell death.

## Graphical abstract

By promoting Caspase‐8 activation within the PANoptosome, NLK modulates pyroptotic, apoptotic, and necroptotic programs in macrophages, thereby driving inflammatory injury and multiorgan damage during sepsis.

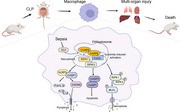

## 1 INTRODUCTION

Sepsis is a life‐threatening syndrome caused by a dysregulated host immune response to infection and is characterised by hyperinflammation, inflammatory cell death, immune dysfunction, and progressive multiorgan failure.^1^, ^2^ These pathological manifestations stem from a profoundly perturbed inflammatory milieu, in which persistent activation of innate immune signalling and excessive release of damage‐associated molecular patterns (DAMPs) fuel tissue injury and perpetuate a self‐reinforcing cycle of inflammation and damage.^3^ This complex pathophysiology underscores the urgent need to define the cellular and molecular mechanisms that amplify inflammation and to identify actionable therapeutic targets in sepsis.

Macrophages serve as key mediators of this dysregulated inflammatory response. During sepsis, they integrate pathogen‐ and danger‐associated signals and subsequently activate multiple inflammatory programmed cell‐death (PCD) pathways—including pyroptosis, apoptosis, and necroptosis—which amplify cytokine production and further disrupt immune homeostasis.^4^, ^5^ A recently defined integrated cell‐death pathway, PANoptosis, functionally coordinates these PCD programs through the assembly of a multiprotein complex termed the PANoptosome, enabling their concerted activation in response to inflammatory stimuli.^6^, ^7^ The PANoptosome is a dynamic supramolecular complex that typically incorporates pattern‐recognition receptors, adaptor proteins (e.g. ASC, FADD), and effector molecules (e.g. Caspase‐1, Caspase‐8, RIPK1, RIPK3, and MLKL). Its precise composition varies depending on the initiating stimuli and cellular context. Within this complex, Caspase‐8 acts as a pivotal molecular switch that integrates upstream signals to determine the dominant cell‐death modality. Upon activation, Caspase‐8 initiates apoptosis via Caspase‐3/7, promotes pyroptosis by cleaving GSDMD either directly or indirectly through Caspase‐1, and suppresses necroptosis through modulation of RIPK1 and RIPK3.^8^, ^9^, ^10^ The coordinated activation of these pathways generates a potent inflammatory cell‐death response that promotes excessive cytokine release and contributes to tissue injury during sepsis.^11^, ^12^ Experimental studies have established PANoptosis as a key pathological driver of sepsis‐induced organ damage, and its pharmacological or genetic inhibition has been shown to markedly attenuate tissue injury, underscoring its therapeutic relevance.^13^, ^14^, ^15^ Despite these advances, the upstream regulatory mechanisms that govern the initiation and modulation of PANoptotic signalling remain incompletely understood. Notably, accumulating evidence suggests that PANoptotic cell death extends beyond the determination of cell fate and may contribute more broadly to the inflammatory milieu within affected tissues.

To identify potential upstream regulators of PANoptosis in sepsis, we analysed single‐cell (GSE167363) and bulk (GSE65682) transcriptomic datasets from peripheral blood mononuclear cells (PBMCs) of patients with sepsis and healthy controls. Among the candidates identified, Nemo‐like kinase (NLK) was significantly upregulated in sepsis, and elevated NLK expression correlated with increased mortality. Consistently, NLK expression correlated with key mediators across PANoptosis programs—pyroptosis (NLRP3, GSDMD), apoptosis (Caspase‐3), and necroptosis (MLKL)—leading us to hypothesise that NLK may function as a regulator integrating or modulating these pathways.

NLK is an evolutionarily conserved serine/threonine kinase within the MAPK family, traditionally implicated in developmental processes and stress responses.^16^ In addition to its catalytic activity, emerging evidence suggests that NLK may also exert regulatory functions that are not strictly dependent on enzymatic signalling,^17^ although the relevance of such non‐canonical properties in inflammatory contexts remains poorly defined. Recent studies have expanded the role of NLK into immune regulation, including modulation of Forkhead box protein P3 (FOXP3) activity in regulatory T cells, CD8^+^ T‐cell differentiation, and antiviral signalling.^18^, ^19^, ^20^ However, whether NLK regulates inflammatory cell‐death programs such as PANoptosis, particularly in the context of sepsis, remains unknown. Here, by integrating multi‐omics analyses with monocyte/macrophage‐targeted NLK conditional knockout models and mechanistic studies, we sought to define the role of NLK in macrophage PANoptosis during sepsis and to elucidate its impact on inflammatory outcomes.

## 2 MATERIALS AND METHODS

### 2.1 Reagents

Primary antibodies against NLK (#94350), Caspase‐8 (#8592), Caspase‐3 (#96662), Caspase‐7 (#9492), MLKL (#37705), p‐MLKL (#37333), RIPK3 (#95702), p‐RIPK3 (#91702S), RIPK1 (#3493), NLRP3 (#15101), GSDMD (#39754), and ASC (#67824) were purchased from Cell Signaling Technology (Danvers, MA, USA). Antibodies against CD68 (28058‐1‐AP), Caspase‐1 (22915‐1‐AP), p‐RIPK1 (66854‐1‐Ig), FADD (P14906‐1‐AP), HA‐Tag (51064‐2‐AP, 66006‐2‐Ig), Flag‐Tag (66008‐4‐Ig, 20543‐1‐AP), and IgG (B900620) were purchased from Proteintech Group, Inc. (Wuhan, China). NLK (sc‐48361), RIPK3 (sc‐374639), and ASC (sc‐514414) antibodies were purchased from Santa Cruz Biotechnology (Santa Cruz, CA, USA). VeriBlot for Immunoprecipitation Detection (ab131366) was obtained from Abcam (Cambridge, UK). Lipopolysaccharide (L4516) was obtained from Sigma‐Aldrich (St. Louis, MO, USA). Recombinant mouse M‐CSF protein (M‐CSF, HY‐P7085) was purchased from MedChemExpress (Monmouth Junction, NJ, USA). DMEM medium (C11995500BT) and foetal bovine serum (10100147) were purchased from Gibco (Thermo Fisher Scientific, Waltham, MA, USA). RPMI‐1640 medium (Cat. no. 31800) was purchased from Solarbio (Beijing, China).

### 2.2 Experimental animals

NLK^fl/fl^ mice on a C57BL/6 background were crossed with Csf1r‐iCre transgenic mice to generate conditional NLK‐deficient mice in Csf1r‐expressing myeloid cells (NLK^fl/fl/Csf1r‐iCre^, NKO). Littermate NLK^fl/fl^ mice lacking the Cre transgene were used as wild‐type (WT) controls. All mouse lines were obtained from GemPharmatech Co., Ltd. (Jiangsu, China). Genotyping was performed by PCR using allele‐specific primer sets to detect the floxed NLK allele and the presence of the Csf1r‐iCre transgene. Detailed genotyping strategies and representative PCR results are provided in Supplementary Information 1. Mice were housed under specific pathogen‐free conditions (22–24°C, 50–65% humidity, 12 h light/dark cycle) with free access to food and water.

### 2.3 Transcriptomic and single‐cell RNA‐seq analysis

Publicly available transcriptomic datasets used in this study were obtained from the Gene Expression Omnibus (GEO) database, and all analyses were conducted in accordance with the original data usage guidelines.

#### 2.3.1 Bulk RNA‐seq dataset (GSE65682)

Whole‐blood transcriptomic profiles from patients with sepsis and healthy controls were retrieved from GEO (accession number: GSE65682). This cohort included samples from both male and female individuals. Raw CEL files were normalised using the robust multi‐array average (RMA) algorithm, followed by probe‐to‐gene symbol mapping and log_2_ transformation. Genes with low expression across samples were filtered out prior to downstream analysis. Given the mixed‐sex composition of the cohort and the study's focus on identifying core sepsis‐associated transcriptional signatures, analyses were performed on the combined dataset without sex‐based stratification. Differential gene expression analysis was conducted using the *limma* package with thresholds of |log_2_ fold change| > .58 and false discovery rate (FDR) < .05. Between‐group comparisons were performed using unpaired Student's *t*‐tests. Pearson's correlation analysis was applied to assess associations between NLK expression and key PANoptotic effectors. Kaplan–Meier survival analyses were performed using the *survminer* package in R.

#### 2.3.2 Single‐cell RNA‐seq dataset (GSE167363)

Single‐cell transcriptomic data derived from peripheral blood mononuclear cells of sepsis patients and healthy controls were obtained from GEO (accession number: GSE167363). After initial data import, quality control was performed using Seurat (v4.3.0). Cells with fewer than 500 or more than 4500 detected genes, mitochondrial transcript proportions exceeding 15%, or haemoglobin gene expression greater than 1% were excluded. Following quality filtering, a total of 49 628 high‐quality cells were retained for downstream analysis. Data were normalised using the LogNormalize method, scaled, and integrated to correct for batch effects. Cell‐type annotation was performed based on established canonical marker genes. To ensure annotation robustness, clustering stability was assessed across multiple resolutions, cluster‐specific marker gene enrichment was examined, and marker expression patterns were visualised using dimensionality reduction, dot plots, and heatmaps. Detailed quality control metrics and annotation validation results are provided in the Supplementary Results.

### 2.4 In vivo experimental design and outcome measures

Male NKO and WT mice (6–8 weeks, 20–22 g) were randomly assigned to sham or cecal ligation and puncture (CLP) groups (*n* = 6–8/group). Sepsis was induced by CLP under pentobarbital anaesthesia (50 mg/kg, intraperitoneally). Postoperative care included subcutaneous administration of saline (50 mL/kg) for fluid resuscitation and buprenorphine (.05 mg/kg) for analgesia. Sham‐operated mice underwent identical laparotomy without ligation or puncture. At 24 h after CLP, mice were euthanised, and blood, lung, liver, kidney, and heart tissues were collected. Outcome measures included survival rate, serum cytokine concentrations, serum biochemical indices, and histopathological injury scores.

### 2.5 Primary macrophage isolation, in vitro experimental design, and outcome measures

Bone marrow–derived macrophages (BMDMs) were obtained from the femurs of male mice. Briefly, bone marrow cells were flushed from femurs, red blood cells were lysed, and 1 × 10^7^ cells per dish were plated in RPMI‐1640 medium supplemented with 10% fetal bovine serum and recombinant M‐CSF (50 ng/mL; MedChemExpress, HY‐P7085). Cells were cultured for 7 days to allow macrophage differentiation before subsequent experiments. Mature BMDMs were stimulated with lipopolysaccharide (LPS, 1 µg/mL, Sigma, L4516) or PBS as control. Spleen‐derived macrophages (SDMs) were isolated from CLP or sham‐operated mice using a lymphocyte separation kit (TBD, LTS1092P) followed by positive selection with anti‐F4/80 MicroBeads (Miltenyi Biotec, 130‐110‐443).

Primary macrophages from wild‐type (WT) and NLK conditional knockout (NKO) mice were divided into four groups: WT‐PBS, WT‐LPS, NKO‐PBS, and NKO‐LPS. Unless otherwise indicated, cells were treated with 1 µg/mL LPS for 12 h. Outcome measures included cytokine release (TNF‐α, IL‐6, IL‐1β, and IL‐18) by enzyme‐linked immunosorbent assay (ELISA); PANoptotic marker expression (NLRP3, cleaved GSDMD, cleaved Caspase‐1/3/7/8, p‐MLKL, p‐RIPK1/3, and FADD) by western blotting and immunofluorescence; ultrastructural changes by transmission electron microscopy (TEM); protein–protein interactions by co‐immunoprecipitation; and interaction domain mapping using truncation constructs.

### 2.6 Histopathological assessment of organ injury by haematoxylin–eosin staining

Formalin‐fixed tissues were paraffin‐embedded, sectioned (5 µm), and stained with haematoxylin–eosin (H&E). Lesion severity was graded using a semiquantitative scoring system (0–3 scale; Table S1). All histological evaluations were performed independently by two blinded investigators.

### 2.7 Immunofluorescence staining

For tissue sections, paraffin‐embedded samples were deparaffinised, rehydrated, subjected to heat‐induced antigen retrieval (Tris‐EDTA buffer, pH 9.0), and blocked with 5% BSA. Sections were incubated with primary antibodies (e.g., anti‐CD68, anti‐Caspase‐8, anti‐ASC) overnight at 4°C, followed by fluorophore‐conjugated secondary antibodies. Nuclei were counterstained with 4′,6‐diamidino‐2‐phenylindole (DAPI). For cultured macrophages, live/dead staining was performed using propidium iodide (PI) and Calcein‐AM (Beyotime, C2015S) according to the manufacturer's protocol. For fixed‐cell immunofluorescence, macrophages were fixed with 4% paraformaldehyde, permeabilised with  .1% Triton X‐100, blocked, and stained with primary antibodies against target proteins, followed by fluorescent secondary antibodies. Images were captured using a confocal laser scanning microscope (Leica TCS SP8).

### 2.8 Immunofluorescence quantification

Immunofluorescence image analysis was performed using ImageJ/Fiji (National Institutes of Health, Bethesda, MD, USA) in a blinded manner. Thresholding was performed using the MaxEntropy algorithm, and background subtraction was applied uniformly across all groups using the same parameters to ensure consistency.

#### 2.8.1 Tissue sections

Macrophages were identified as CD68^+^DAPI^+^ cells. For each image, a CD68‐based region‐of‐interest (ROI) mask was created to confine analysis to the macrophage compartment. Colocalisation was quantified using two complementary strategies tailored to the signal characteristics of each marker. For area‐distributed markers, including GSDMD‐N, cleaved caspase‐3, and p‐MLKL, colocalisation was expressed as the percentage of CD68^+^ area co‐positive for the respective marker, calculated as (double‐positive area / total CD68^+^ area) × 100%.

For punctate structures, such as ASC and Caspase‐8, a Manders‐type overlap metric was applied. Puncta masks for each channel were generated using the MaxEntropy auto‐thresholding algorithm in Fiji, and the overlap ratio was calculated as (overlapping signal area / CD68^+^ area) × 100%.

For quantification of total macrophage abundance, CD68^+^ signal was segmented using a fixed intensity threshold, and total CD68^+^ area per field, used as a surrogate measure of macrophage abundance, was measured. Values were normalised to the mean of the WT‐Sham group and expressed as fold change.

For assessment of macrophage polarisation, CD86 or CD163 signals were quantified within the CD68^+^ ROI. Polarised macrophage subsets were expressed as the percentage of CD68^+^ area co‐positive for CD86 or CD163, respectively, calculated as (CD68^+^CD86^+^ or CD68^+^CD163^+^ area / total CD68^+^ area) × 100%.

Three random, non‐overlapping fields were analysed per animal. The average value per animal was used as a single data point for statistical comparisons (*n* = 6 animals per group).

#### 2.8.2 Primary BMDMs

For colocalisation analysis of the RIPK3–ASC–Caspase‐8 signalling complex, a Manders‐type overlap metric was applied. Puncta masks were generated using the MaxEntropy algorithm, and the colocalisation ratio was calculated as (overlap area / reference channel area) × 100%. Three random fields per well were analysed, with the average value per well used for statistical comparisons.

For colocalisation analysis of NLK and Caspase‐8, regions of interest (ROIs) encompassing individual cells were manually defined, and colocalisation was quantified by calculating Pearson's correlation coefficient (Pearson's *R*) based on pixel intensity values within each ROI. Background fluorescence was subtracted prior to analysis. Pearson's *R* values were calculated for multiple cells per condition, and the mean value per condition was used for statistical comparisons.

For Calcein‐AM/PI staining, total cells were identified by DAPI staining. Live (Calcein‐AM^+^) and dead (PI^+^) cells were quantified based on fluorescent intensity. Cell death rate was calculated as (number of PI^+^ cells) / (number of DAPI^+^ cells) × 100%. Three random fields were analysed per well, and the average death rate per well constituted one independent experiment.

### 2.9 ELISA and biochemical assay

Serum and cell supernatants were analysed for tumour necrosis factor‐alpha (TNF‐α; ml002095), interleukin‐6 (IL‐6; IC50325‐1), interleukin‐1 beta (IL‐1β; E‐EL‐R1327c), interleukin‐18 (IL‐18; CK‐E20324), and cardiac troponin I (cTnI; ml001932) using commercial ELISA kits (Shanghai Enzyme‐linked Biotechnology Co., Ltd., Shanghai, China), according to the manufacturer's instructions. Serum alanine aminotransferase (ALT), total bilirubin, blood urea nitrogen (BUN), creatinine, and lactate dehydrogenase (LDH) were measured using an automated biochemical analyser (BS‐240VET, Mindray, Shenzhen, China).

### 2.10 Protein extraction and western blotting

Proteins were extracted using RIPA buffer (Beyotime Biotechnology) supplemented with protease inhibitor cocktail (Roche, 04693116001) and phosphatase inhibitor cocktail (Roche, 04906837001). Protein concentrations were determined using a BCA assay kit (CoWin Bio., China). Equal amounts of protein were resolved by SDS‐PAGE (Zhonghui Hecai Biomedical Technology Co., Ltd., PE008) and transferred onto PVDF membranes (Millipore, Cat. no. ISEQ00010). After blocking with 5% non‐fat milk in TBST, membranes were incubated with primary antibodies overnight at 4°C, followed by HRP‐conjugated secondary antibodies. Immunoreactive bands were detected using enhanced chemiluminescence reagents.

### 2.11 Caspase‐8 and Caspase‐3 enzymatic activity assays

Caspase‐8 and Caspase‐3 activities were measured using colorimetric assay kits (Beyotime Biotechnology, C1151 and C1115) according to the manufacturer's instructions. Briefly, cell lysates were prepared in the supplied lysis buffer and cleared by centrifugation. Equal amounts of protein were incubated with the caspase‐specific chromogenic substrates Ac‐IETD‐pNA (for Caspase‐8) or Ac‐DEVD‐pNA (for Caspase‐3) in reaction buffer at 37°C. After incubation, the release of p‐nitroaniline (pNA) was quantified by measuring absorbance at 405 nm using a microplate reader. Caspase activity was normalised to total protein content and expressed relative to control conditions.

### 2.12 Transmission electron microscopy (TEM) analysis of cell death–associated ultrastructural features

Macrophages were fixed in 2.5% glutaraldehyde in  .1 M phosphate buffer (pH 7.4) at 4°C overnight, post‐fixed in 1% osmium tetroxide for 1 h, dehydrated through a graded ethanol series, and embedded in epoxy resin. Ultrathin sections were cut with an ultramicrotome, stained with uranyl acetate and lead citrate, and examined using a transmission electron microscope (Hitachi HT7800, Tokyo, Japan). Representative imageswere acquired with a digital camera system.

TEM was used to visualise ultrastructural features associated with distinct programmed cell‐death pathways and to provide supportive morphological evidence, rather than to serve as a definitive classification of cell‐death modalities. Image analysis was performed by two independent investigators in a blinded manner. Based on previously established ultrastructural criteria,^21^, ^22^ cells displaying predominant morphological features consistent with pyroptosis (cell swelling with largely preserved organelles and membrane pore formation), apoptosis (cell shrinkage, chromatin condensation, and nuclear fragmentation), or necroptosis (organelle swelling, vesiculation, and plasma membrane rupture) were recorded. Cells exhibiting ambiguous, overlapping, or mixed ultrastructural features were excluded from quantification and were not assigned to a specific death category. For quantitative analysis, 100 macrophages per group were evaluated across samples derived from three independent mice or experiments, and the relative proportions of cells exhibiting each predominant ultrastructural phenotype were calculated.

### 2.13 Molecular docking

The three‐dimensional structures of NLK and caspase‐8 were obtained from the Protein Data Bank (PDB) or predicted using AlphaFold (https://alphafold.ebi.ac.uk). Protein–protein docking was performed using ClusPro 2.0 (https://cluspro.bu.edu/), and the top‐ranked models were analysed. Predicted interaction interfaces were visualised and annotated using PyMOL (Schrödinger, LLC). Docking scores, hydrogen bonds, and interface residues were analysed to predict potential binding modes.

### 2.14 Cell transfection and overexpression

Caspase‐8 overexpression in BMDMs was achieved using an adenoviral vector encoding murine Caspase‐8 (GeneChem, Shanghai, China) for rescue experiments. For heterologous expression studies, HEK293T cells were transiently transfected with pCMV‐NLK‐3×HA, pCMV‐Caspase‐8‐3×FLAG, or the corresponding truncated mutants (Miaoling Biotechnology, Wuhan, China) using BeyoPEI™ reagent (Beyotime, C0541) according to the manufacturer's instructions. Transfection efficiency was verified by immunoblotting.

### 2.15 Co‐immunoprecipitation (Co‐IP)

For endogenous Co‐IP assays, BMDMs and SDMs were lysed under non‐denaturing conditions, and clarified lysates were incubated overnight at 4°C with antibodies against FADD or RIPK1, followed by precipitation with Protein A/G magnetic beads (Bioinkedin, IK‐1004). For Heterologous Co‐IP assays, HEK293T cells were lysed 72 h post‑transfection. Cleared lysates were incubated overnight at 4°C with Anti‑HA or Anti‑FLAG magnetic beads (Bioinkedin, IK‐1009, IK‐1011). Immunoprecipitated complexes were washed extensively and analysed by SDS‐PAGE and immunoblotting.

### 2.16 Statistical analysis

All data are expressed as the mean ± standard deviation (SD) unless otherwise specified. Statistical analyses were performed using GraphPad Prism 8.0 (GraphPad Software, San Diego, CA, USA). Data distribution was assessed using the Shapiro–Wilk normality test, and homogeneity of variance was evaluated using Levene's test. For comparisons between two groups, the unpaired two‑tailed Student's *t*‑test was used for normally distributed data; whereas the Mann–Whitney U test was applied for non‐normally distributed data. For comparisons among multiple groups, parametric data were analysed by one‑way ANOVA, followed by Bonferroni's post hoc test if the overall ANOVA was significant; otherwise, the Kruskal–Wallis test with Dunn's post hoc test was used. A *p* value < .05 was considered statistically significant.

## 3 RESULTS

### 3.1 NLK elevation correlates with poor survival and PANoptosis genes in sepsis

**FIGURE 1:**
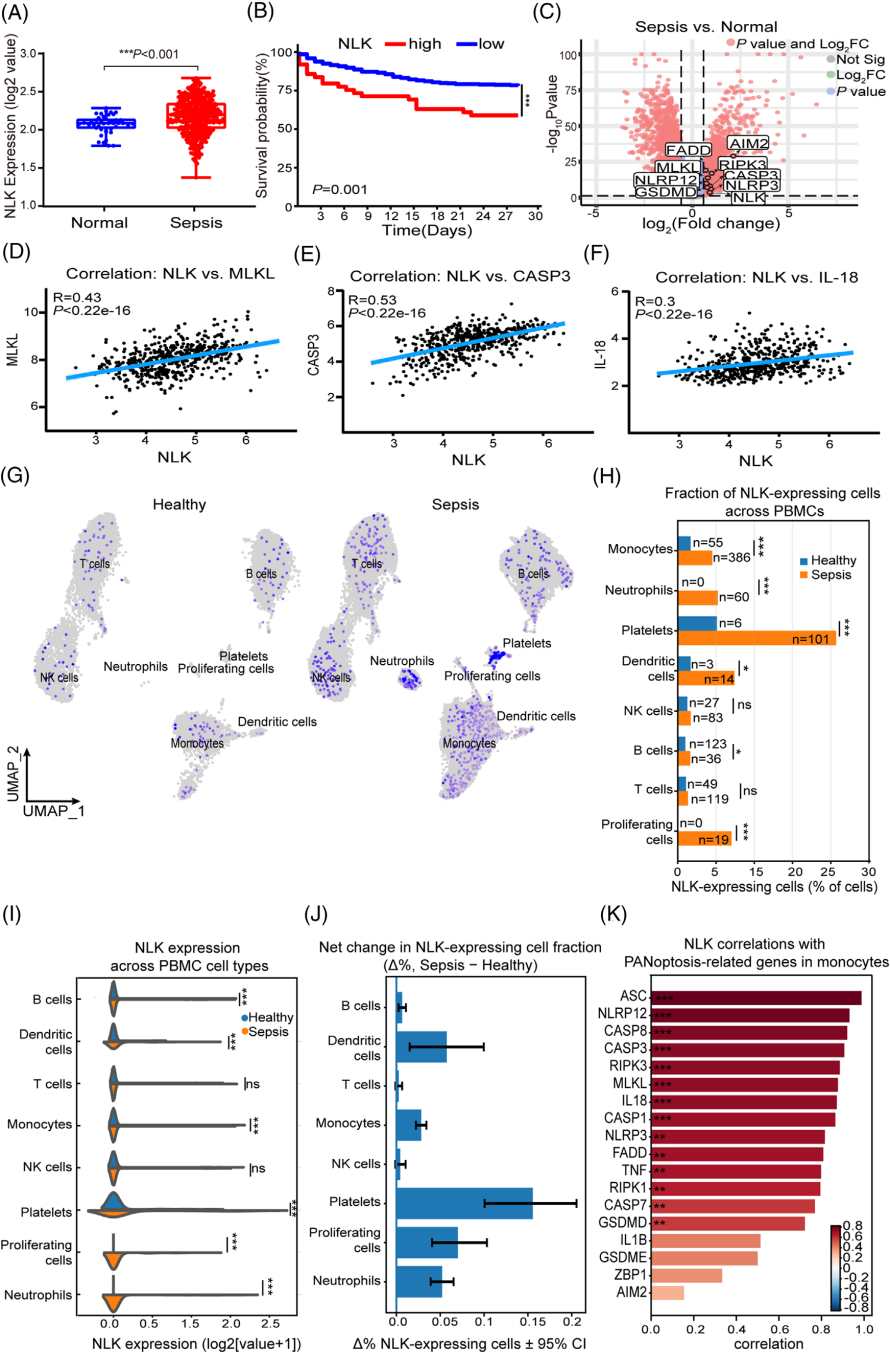
NLK elevation correlates with poor survival and PANoptosis genes in sepsis. (A) NLK mRNA expression in whole blood from GSE65682 cohort (sepsis, *n* = 760; healthy, *n* = 42), log_2_‑transformed; unpaired *t‑*test. (B) Kaplan–Meier 28‑day survival curves stratified by NLK expression using a maximally selected rank statistic cut‐off (NLK‐high, *n* = 49; NLK‐low, *n* = 430); log‑rank test. (C) Volcano plot of differentially expressed genes in sepsis vs control (|log_2_FC| > 0.58, FDR‐adjusted *p *< .05), which highlights NLK and selected PANoptosis genes. (D–F) Pearson's correlations analyses between NLK and MLKL (D), CASP3 (E) or IL‑18 (F) in sepsis whole blood samples; the lines show linear regression with 95% confidence intervals. (G) UMAP visualisation of eight immune cell populations identified from PBMC single‐cell RNA‐seq data of healthy controls and patients with sepsis (GSE167363). NLK‐expressing cells are highlighted. (H) Fraction of NLK‐expressing cells across PBMC subsets in healthy controls and patients with sepsis. Group comparisons were performed using Fisher's exact test at the cell level. Numbers within bars indicate absolute counts of NLK‐positive cells. (I) Violin plots showing NLK expression distributions across PBMC cell types in healthy and septic conditions. Group comparisons were performed using the Mann–Whitney U test. Expression values are shown as log2(value + 1). (J) Net change in the fraction of NLK‐expressing cells between sepsis and healthy conditions (Δ% NLK‐expressing cells). Error bars represent 95% confidence intervals estimated by bootstrap resampling. (K) Spearman's correlation analysis between NLK expression and representative PANoptosis‐related genes within the monocyte population. For non‐normally distributed single‐cell expression data, non‐parametric tests were applied as indicated. Statistical significance is indicated as follows: *p < .05, **p < .01, ***p < .001.

To identify potential regulators of sepsis, we first analysed the bulk blood transcriptomic dataset GSE65682, which included 760 patients with sepsis and 42 healthy controls. NLK expression was significantly higher in patients with sepsis than that in controls (Figure 1A). Stratification of patients into NLK‐high and NLK‐low groups revealed that elevated NLK was associated with a markedly reduced 28‐day survival rate (59.2% vs. 78.1%, log‐rank *p* < .01, Figure 1B). In this large cohort, differential expression analysis identified multiple genes in PANoptotic pathways that were upregulated alongside NLK (Figure 1C). Moreover, NLK expression correlated positively with representative effectors, such as MLKL, Caspase‐3, and IL‐18 (Figure 1D–F). Together, these findings implicate NLK in septic mortality and inflammatory cell death.

To define the cellular basis of NLK dysregulation, we next analysed single‐cell RNA‐seq data from PBMCs (GSE167363). Unsupervised UMAP clustering identified eight major PBMC populations, including T cells, B cells, NK cells, monocytes, neutrophils, dendritic cells, platelets, and proliferating cells (Figure S1A). Whereas NLK expression was sparse under homeostatic conditions, sepsis triggered a marked expansion of NLK‐expressing cells across multiple immune lineages (Figure 1G). Quantitative analysis revealed that although platelets exhibited the highest within‐lineage proportion of NLK‐positive cells, monocytes constituted the largest absolute reservoir of NLK‐expressing cells in sepsis, underscoring their quantitative dominance in the NLK response (Figure 1H). At the single‐cell level, NLK expression remained low or undetectable in most cells; however, immune subsets with increased NLK positivity displayed a consistent rightward shift in expression distributions (Figure 1I), indicating that NLK dysregulation is driven primarily by cellular expansion rather than uniform transcriptional upregulation. This effect was further quantified as a robust increase in the proportion of NLK‐positive cells (Δ% expressing) across several immune lineages (excluding proliferating cells), most pronounced in platelets and dendritic cells (Figure 1J). Together, these data establish NLK as a broadly inducible, sepsis‐responsive gene. Notably, however, only within the monocyte compartment did NLK expression exhibit strong and specific positive correlations with a core network of PANoptosis execution genes, including *ASC*, *CASP8*, *RIPK3*, *MLKL*, and *NLRP3* (Figure 1K; see also Figure S1B). This cell‐type–specific functional coupling, together with the quantitative predominance of monocytes and their role as precursors of tissue‐resident macrophages^23^, ^24^—a canonical cellular model for PANoptosis—provides a compelling rationale to focus subsequent mechanistic investigations on macrophage PANoptosis.

Collectively, these analyses demonstrate that NLK is induced in sepsis, enriched within the monocyte–macrophage axis, and transcriptionally linked to PANoptosis, thereby justifying its in‐depth investigation in macrophage cell death.

### 3.2 NLK deficiency attenuates organ injury and improves survival in a murine sepsis model

**FIGURE 2:**
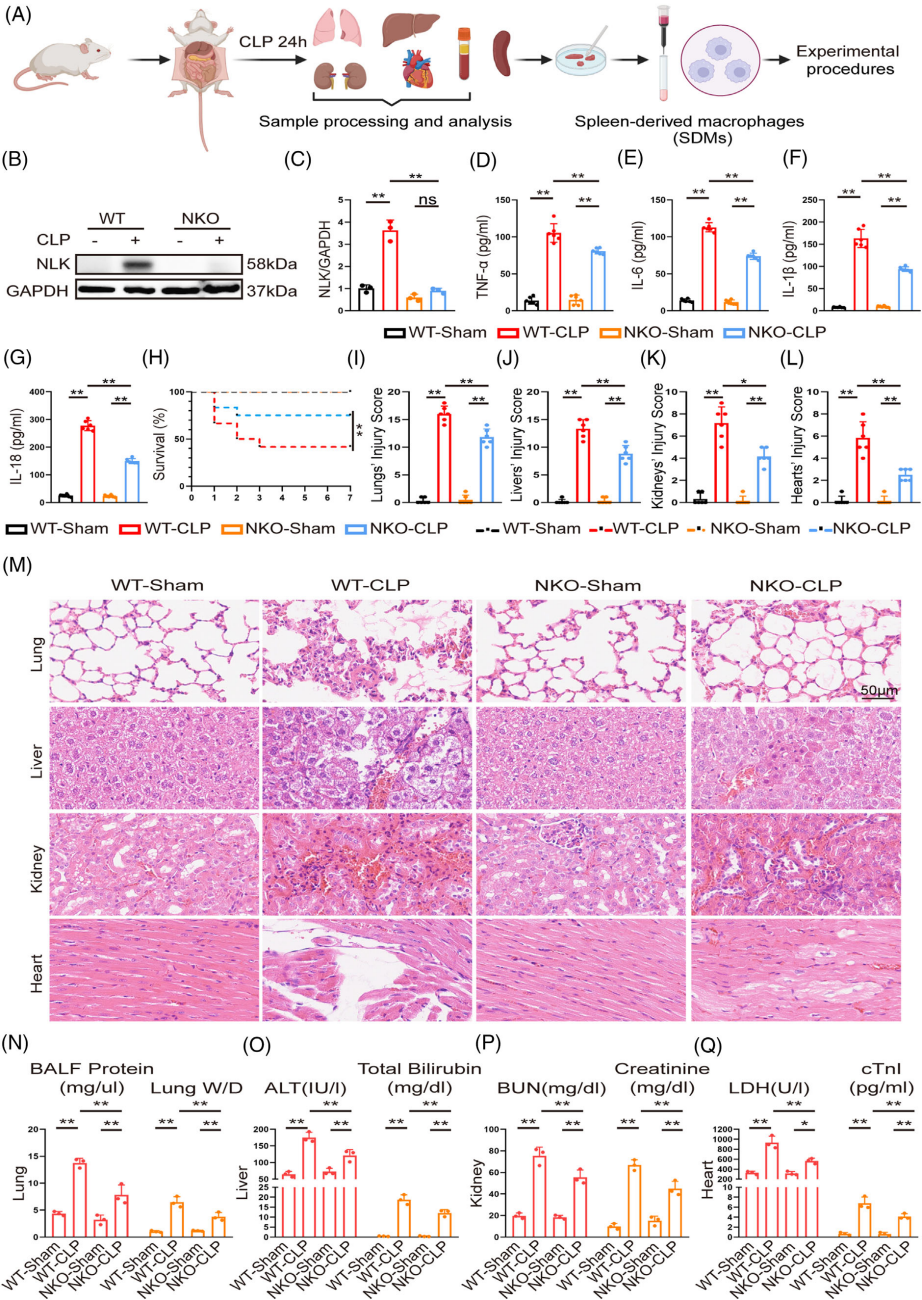
NLK deficiency attenuates organ injury and improves survival in a murine sepsis model. (A) Experimental design of the CLP model in NLK conditional knockout mice driven by Csf1r‐iCre (NKO) and wild‐type (WT) mice. (B, C) Western blot analysis of NLK expression in SDMs (*n* = 3 independent biological replicates). (D–G) Serum TNF‐α, IL‐6, IL‐1β, and IL‐18 measured by ELISA (*n* = 6 mice per group, independent biological replicates). (H) Kaplan–Meier survival analysis over 7 days post‐CLP (*n* = 12 mice per group, independent biological replicates), **p < .01 by log‐rank test. (I–M) Histopathological injury scores and representative H&E‐stained sections of lung, liver, kidney, and heart (scale bar: 100 µm; *n* = 6 mice per group). (N–Q) Quantification of organ injury markers: BALF protein concentration and wet/dry lung weight (lungs); ALT and total bilirubin (liver); BUN and creatinine (kidneys); LDH and cTnI (heart) (*n* = 3 independent biological replicates). Statistical differences were analysed by one‑way ANOVA with Bonferroni's post hoc test, *p < .05 and **p < .01.

To investigate the functional role of NLK in sepsis, we employed a CLP model in NLK conditional knockout mice driven by Csf1r‐iCre (Figure 2A). Consistent with the low basal expression of NLK, its levels were minimal in macrophages under homeostatic conditions, and no overt differences were observed between WT and NLK‐deficient mice at baseline. Upon septic challenge, however, CLP markedly increased NLK protein expression in WT mice, accompanied by elevated systemic levels of TNF‐α, IL‐6, IL‐1β, and IL‐18 (Figure 2B–G). In contrast, NLK deficiency substantially reduced inflammatory cytokine release and significantly improved survival, increasing the 7‐day survival rate from 41.7% in WT mice to 75% in NKO mice (log‐rank test, *p *< .05, Figure 2H). Histopathological analysis revealed that sepsis‐associated injury in the lung, liver, kidney, and heart was markedly attenuated in NKO mice (Figure 2I–M). Consistently, multiple indices of organ function—including BALF protein content, lung wet/dry weight ratio, serum ALT, total bilirubin, BUN, creatinine, LDH, and cTnI—were significantly improved in NKO mice compared with that in WT controls (Figure 2N–Q). Collectively, these results indicate that NLK deficiency confers robust protection against systemic inflammation, multiorgan injury, and mortality during polymicrobial sepsis.

### 3.3 NLK deficiency shifts the execution of sepsis‐associated PANoptosis towards necroptosis

**FIGURE 3:**
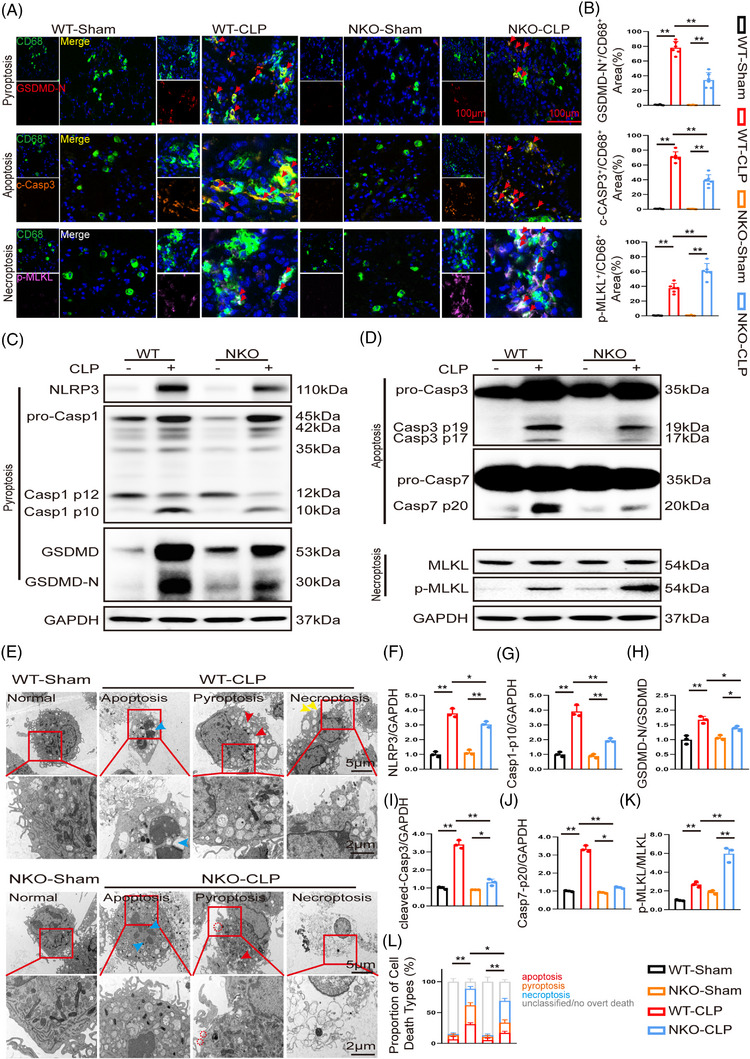
NLK deficiency shifts the execution of sepsis‐associated PANoptosis towards necroptosis. (A, B) Immunofluorescent co‐staining and quantification of lung sections showing CD68^+^ pulmonary macrophages (green) co‐labelled with GSDMD‐N (red, pyroptosis), cleaved caspase‐3 (orange, apoptosis), and p‐MLKL (purple, necroptosis) (scale bar: 100 µm; *n* = 6 mice per group). (C, D) Representative immunoblotting analyses of pyroptosis‐related proteins (NLRP3, Caspase‐1, GSDMD), apoptosis‐related proteins (cleaved Caspase‐3/7), and necroptosis‐related proteins (MLKL, p‐MLKL) in SDMs from each group. (E) Representative TEM images illustrating macrophages displaying ultrastructural features consistent with pyroptosis, apoptosis, or necroptosis following CLP challenge. Blue arrows indicate vesicle formation; red circles mark membrane pore formation; yellow arrows highlight plasma membrane rupture and translucent cytoplasm (scale bar: 0.1 µm). (F–K) Quantification of immunoblot signals from panels C and D (*n* = 3 independent biological replicates). (L) Relative proportions of macrophages exhibiting predominant death‐associated ultrastructural features consistent with pyroptosis, apoptosis, or necroptosis, as assessed by TEM. Each bar represents the distribution of ultrastructural phenotypes among 100 macrophages analysed per sample (*n* = 3 independent biological replicates); cells lacking overt death‐associated features or displaying ambiguous or mixed morphology were counted as unclassified. *p < .05 and **p < .01 by one‐way ANOVA with Bonferroni's post hoc test.

Immunofluorescence analysis of lung sections demonstrated that CLP triggered concurrent activation of pyroptosis, apoptosis, and necroptosis in pulmonary macrophages, as shown by co‑staining of CD68 with GSDMD‑N, cleaved Caspase‑3, and p‑MLKL (Figure 3A and B). Consistent with this, immunoblotting revealed marked upregulation of NLRP3, caspase‐1 p10, GSDMD‐N, cleaved Caspase‐3/7, and p‐MLKL in WT mice following CLP (Figure 3C, D, and F–K). TEM analysis provided supportive morphological evidence of macrophages displaying ultrastructural features associated with all three programmed cell‐death pathways, which complemented, but did not replace, the classification based on pathway‐specific molecular markers, consistent with previous reports (Figure 3E and L).^14^, ^22^ In contrast, NLK deficiency markedly reduced pyroptosis‐ and apoptosis‐associated markers while simultaneously enhancing p‐MLKL expression and staining, indicating a preferential shift towards necroptosis (Figure 3D and K). Correspondingly, NLK‐deficient macrophages more frequently exhibited necroptosis‐associated ultrastructural features by TEM (Figure 3E and L). Notably, this phenotypic shift was not attributable to global macrophage depletion or altered recruitment, as the overall CD68^+^ macrophage abundance increased comparably in WT and NLK‐deficient lungs following CLP (Figure S1C and D). Instead, NLK deficiency reshaped the functional macrophage landscape, skewing the population from a CD68^+^CD86^+^ (M1‑like) towards a CD68^+^CD163^+^ (M2‑like) phenotype following CLP (Figure S1E and F).

These observations indicate that NLK deficiency reprograms macrophage PANoptosis in sepsis, attenuating pyroptotic and apoptotic components while favouring the necroptotic pathway. Given the central role of Caspase‐8 in promoting apoptosis/pyroptosis and restraining RIPK1/RIPK3‐mediated necroptosis, we hypothesised that impaired Caspase‑8 activation may underlie this death‑mode switch in NLK‑deficient macrophages.

### 3.4 NLK facilitates Caspase‐8 activation and limits necroptotic signalling

**FIGURE 4:**
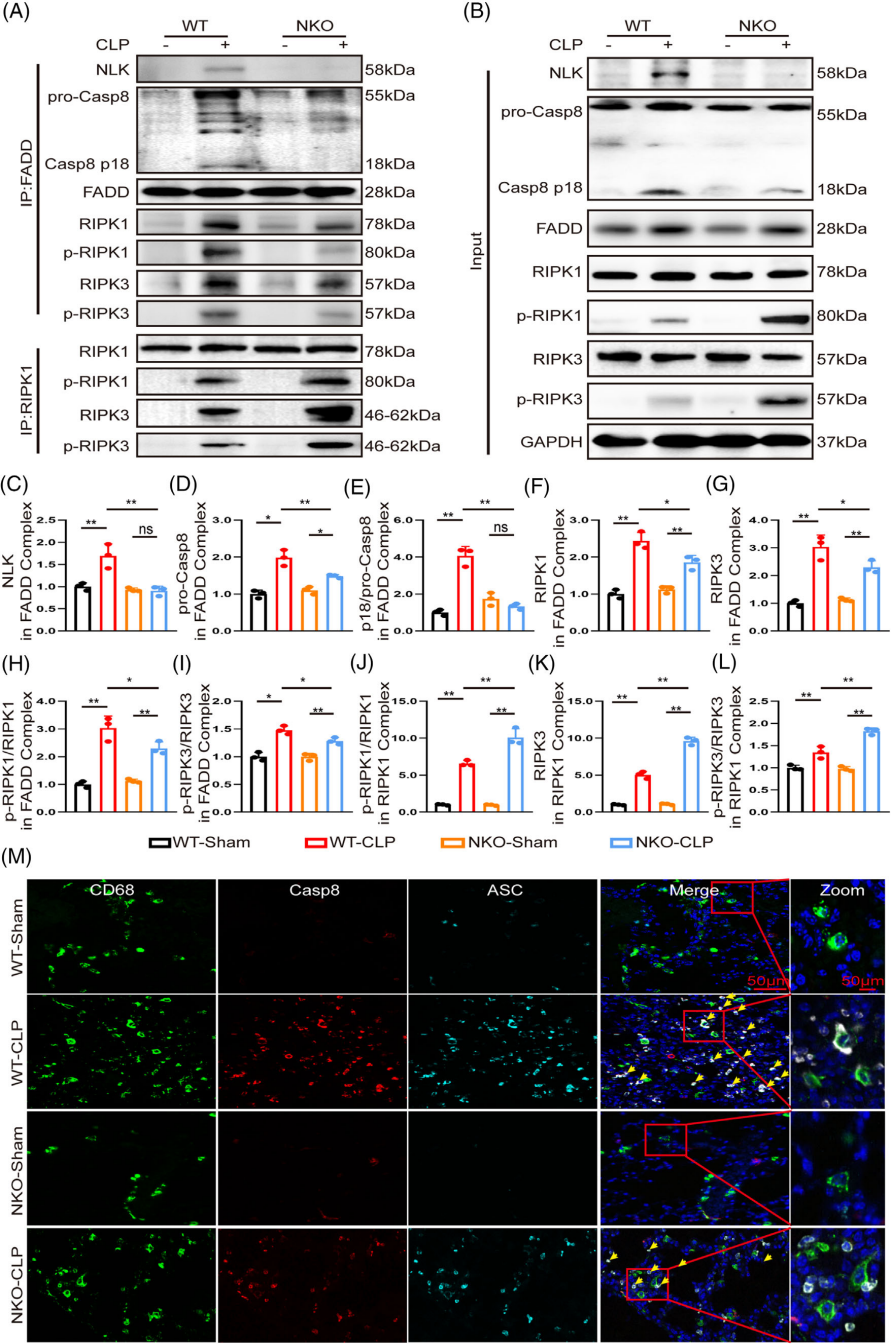
NLK deficiency disrupts PANoptosome assembly and augments RIPK1/3‐ dependent necrosome formation in vivo. (A, B) Representative immunoblots of FADD‐ and RIPK1‐associated complexes, together with corresponding input samples, in macrophages isolated from WT and NKO mice at 8 h post‐CLP. Co‐immunoprecipitates were probed for NLK, Caspase‐8, FADD, RIPK1, p‐RIPK1, RIPK3, and p‐RIPK3. (C–I) Quantification analysis of Caspase‐8, FADD, RIPK1, p‐RIPK1, RIPK3, and p‐RIPK3 in FADD‐associated complexes (*n* = 3 independent biological replicates). (J–L) Quantification analysis of p‐RIPK1, RIPK3, and p‐RIPK3 in RIPK1‐associated complexes (*n* = 3 independent biological replicates). (M) Representative confocal immunofluorescence images of lung macrophages stained for CD68 (green), Caspase‑8 (red), and ASC (cyan). Merged images indicate ASC–Caspase‐8 colocalisation; boxed regions show magnified views. Scale bars: 50 µm. Statistical significance was determined by using one‑way ANOVA with Bonferroni's post hoc test; *p < .05, **p < .01 and ns indicates *p *> .05.

To test this hypothesis, we examined the assembly and activation status of the molecular complex governing PANoptosis. Previous studies have established Caspase‐8, FADD, and RIPK1/3 as core components of the PANoptosome orchestrating PANoptosis.^25^, ^26^ In WT macrophages, CLP stimulation promoted Caspase‐8 cleavage accompanied by modest phosphorylation of RIPK1 and RIPK3, whereas NLK deficiency markedly attenuated Caspase‐8 activation but amplified RIPK1/3 phosphorylation (Figures 4B and S1G–I). Although CLP stimulation increased the expression of the adaptor protein FADD, its abundance remained comparable between WT and NKO macrophages (Figures 4B and S1J). We next evaluated Caspase‐8 activation within FADD‐containing complexes. Upon CLP stimulation, WT macrophages showed efficient recruitment and effective activation of Caspase‐8, as reflected by a high ratio of the cleaved p18 fragment to full‐length pro‐Caspase‐8 in FADD immunoprecipitates (Figure 4A and E). In NLK‐deficient macrophages, Caspase‐8 recruitment into FADD complexes was reduced but not abolished; however, its activation was ineffective, as the p18/pro‐Caspase‐8 ratio failed to increase above basal levels. These findings indicate a specific defect in Caspase‐8 activation within FADD‐containing complexes in the absence of NLK, despite partial preservation of Caspase‐8 recruitment. We next examined RIPK1‐containing complexes. Although total cellular levels of RIPK3 did not show a parallel increase upon CLP stimulation, the amount of RIPK3 and p‐RIPK3 recovered in RIPK1 immunoprecipitates were increased following CLP challenge, with a further augmentation observed in NLK‐deficient macrophages (Figure 4A and J–L). This complex‐associated enrichment reflects enhanced recruitment and activation of RIPK3 within RIPK1‐containing complexes, rather than changes in overall RIPK3 abundance. This pattern is consistent with a shift towards necrosome assembly and necroptotic signalling. Consistent with previous reports defining PANoptosome‐associated signalling, ASC–Caspase‐8 colocalisation was used as a representative spatial readout of PANoptosome engagement. Accordingly, CLP robustly induced ASC–Caspase‐8 colocalisation in WT macrophages, whereas this interaction was markedly reduced in NLK‐deficient cells, further supporting impaired PANoptosome assembly^6^, ^27^, ^28^ (Figures 4M and S1K). These results show that NLK promotes Caspase‐8 activation within PANoptosome‐associated complexes and limits RIPK1/3‐dependent necrosome formation.

### 3.5 NLK regulates Caspase‐8–dependent execution of LPS‐induced PANoptosis in macrophages

**FIGURE 5:**
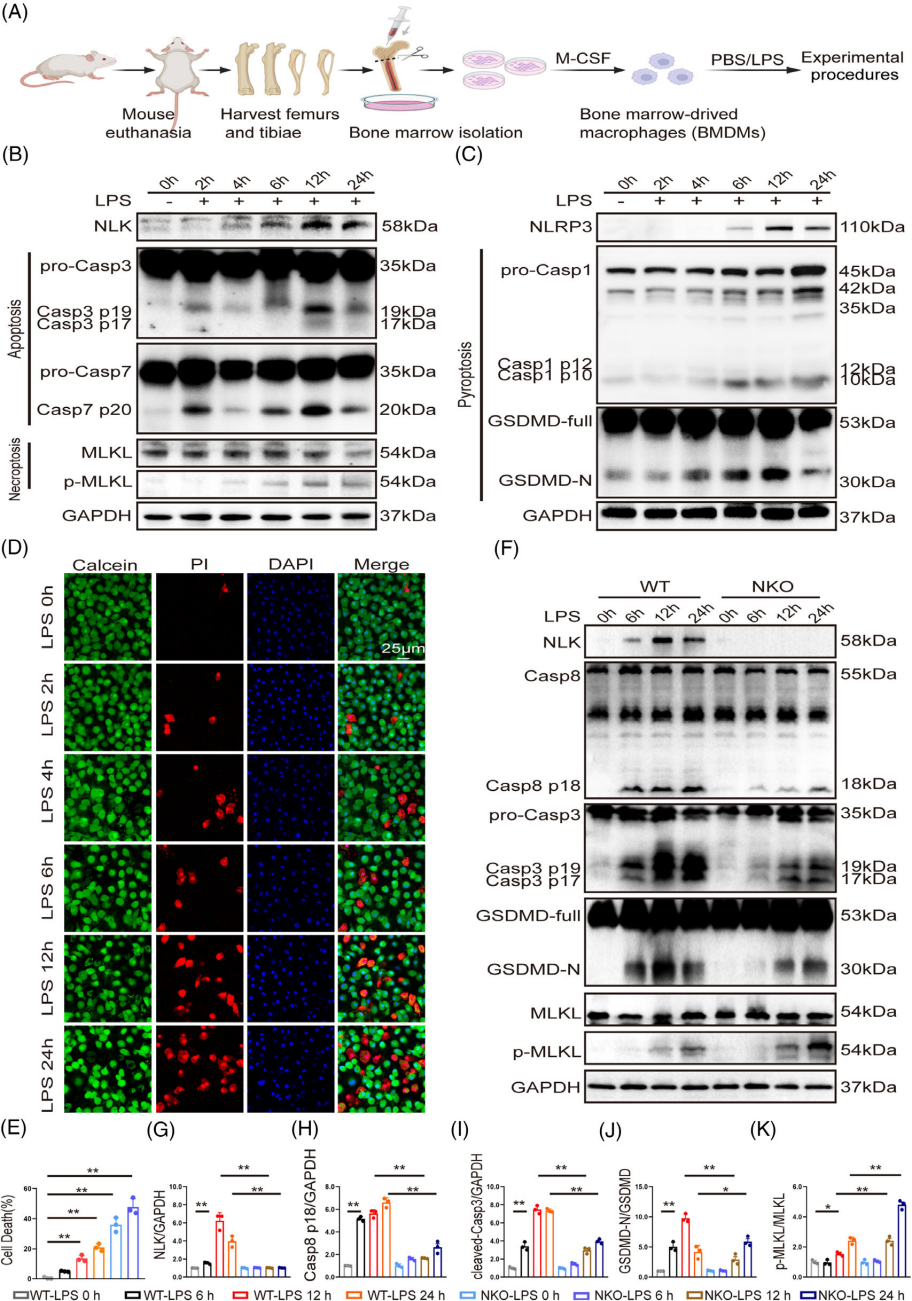
LPS stimulation induces PANoptosis in BMDMs. (A) Experimental scheme for BMDMs generation and LPS stimulation. (B, C) Representative time‐course immunoblot analysis of NLK and PANoptosis‐related markers, including apoptosis (cleaved Caspase‐3, Caspase‐7), necroptosis (MLKL, p‐MLKL), and pyroptosis (NLRP3, pro‐/cleaved Caspase‐1, full‐length/N‐terminal GSDMD) in WT BMDMs following LPS treatment. (D, E) PI/calcein AM staining of BMDMs at the indicated time points (calcein AM for viable cells, green; PI for dead cells, red; scale bar: 100 µm) and quantitative analysis (*n* = 3 per group, independent biological replicates). (F) Time‐course immunoblot analysis of NLK, Caspase‐8, cleaved Caspase‐3, and full‐length/N‐terminal GSDMD in WT and NKO BMDMs following LPS treatment. (G–K) Quantitative analysis of NLK, Caspase‐8, cleaved Caspase‐3, and full‐length/N‐terminal GSDMD in panel F (*n* = 3 independent biological replicates). Statistical significance was determined using one‑way ANOVA with Bonferroni's post hoc test; *p < .05 and **p < .01.

To determine whether the in vivo phenotype was recapitulated under defined inflammatory conditions, WT and NLK‐deficient BMDMs were stimulated with LPS (Figure 5A), a stimulus previously shown to activate PANoptosis or its component pathways in multiple immune cell types.^22^, ^29^ Time‐course analysis (0–24 h) in WT BMDMs revealed that LPS induced a progressive and coordinated activation of PANoptosis‐associated signalling. This response was characterised by time‐dependent cleavage of Caspase‐3/7, phosphorylation of MLKL, and induction of NLRP3, Caspase‐1, and GSDMD (Figures 5A and B and S1H–N). These molecular events were accompanied by a gradual increase in cell death (Figure 5D and E) and defined a critical activation window between 6 and 12 h, with maximal PANoptotic signalling observed at approximately 12 h.

To assess whether NLK regulates LPS‐induced PANoptotic signalling through Caspase‐8, we next compared the activation kinetics of key death pathways in WT and NLK‐deficient BMDMs. In WT cells, LPS stimulation induced robust and time‐dependent cleavage of Caspase‐8, which coincided with activation of downstream apoptotic (cleaved Caspase‐3), pyroptotic (GSDMD‐N), and necroptotic (p‐MLKL) markers (Figure 5F–K). In contrast, NLK deficiency exhibited markedly reduced Caspase‐8 cleavage throughout the time course. This attenuation was accompanied by diminished activation of cleaved Caspase‐3 and GSDMD‐N, whereas phosphorylation of MLKL became more pronounced at later time points (Figure 5F–K). Together, these kinetic differences indicate that NLK deficiency alters the execution balance of PANoptotic signalling, favouring necroptotic engagement as Caspase‐8 activity declines.

### 3.6 NLK deficiency favours a necroptosis‑associated phenotype during LPS‑induced macrophage death

To determine how these kinetic alterations translate into terminal outcomes, we analysed inflammatory mediator release and death‐execution patterns at 12 h post‐LPS, when PANoptotic markers showed robust activation. Analysis of secreted mediators revealed a profile consistent with a shift in death modality. Compared to WT controls, NLK‐deficient macrophages produced significantly lower levels of TNF‐α, IL‐6, IL‐1β, and IL‐18—cytokines commonly associated with pyroptotic and apoptotic responses (Figure S1O–R). In contrast, secretion of CXCL10, a chemokine linked to MLKL activation and necroptotic signalling,^30^ was markedly increased in NLK‐deficient macrophages (Figure S1S).

**FIGURE 6:**
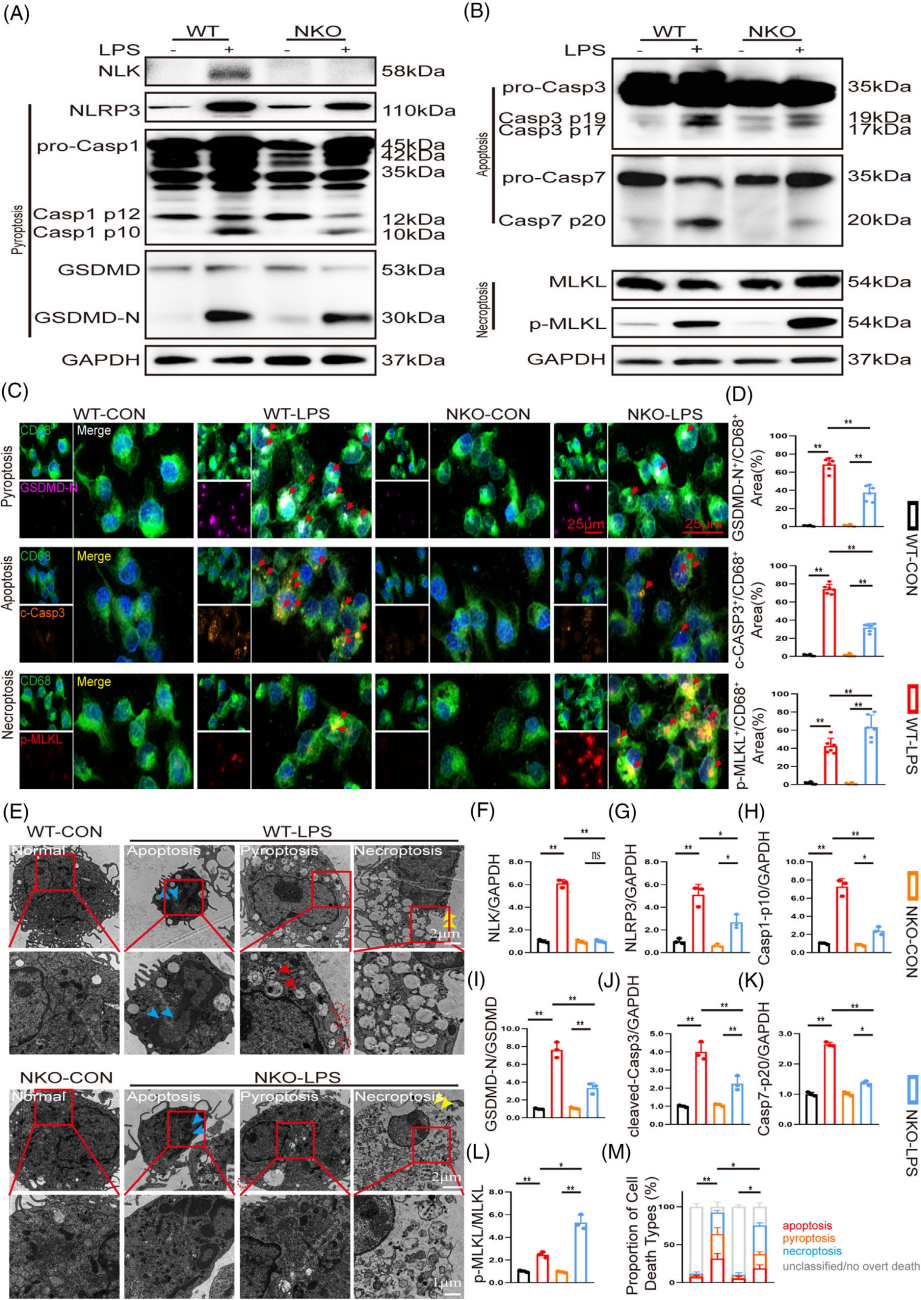
NLK deficiency shifts LPS‐induced PANoptosis towards necroptosis in BMDMs. (A, B) Representative immunoblots of pyroptosis (NLRP3, Caspase‐1, GSDMD), apoptosis (Caspase‐3, Caspase‐7), and necroptosis (MLKL, p‐MLKL) markers in PBS/LPS‐treated BMDMs. (C, D) Immunofluorescent co‐staining and quantification of CD68 (green) with GSDMD‐N (purple, pyroptosis), cleaved caspase‐3 (orange, apoptosis), and p‐MLKL (red, necroptosis) in BMDMs from each group. Scale bar: 100 µm; *n* = 3 independent biological replicates. (E) Representative TEM images of BMDMs showing the ultrastructural features consistent with pyroptosis, apoptosis, and necroptosis. Blue arrows indicate chromatin condensation and nuclear shrinkage (apoptosis); red arrows and circles denote cellular swelling, membrane pore formation and vesiculation (pyroptosis); yellow arrows indicate plasma membrane rupture and translucent cytoplasm (necroptosis). Scale bar: 0.1 µm. (F–L) Quantification of immunoblot signals from panels A and B. (M) Relative proportions of BMDMs exhibiting predominant apoptosis‐, pyroptosis‐, or necroptosis‐associated ultrastructural features based on TEM. Each bar represents the distribution of ultrastructural phenotypes among 100 macrophages analysed per sample from *n* = 3 independent biological replicates; cells lacking overt death‐associated features or displaying ambiguous morphology were classified as unclassified. Statistical differences were analysed by one‑way ANOVA with Bonferroni's post hoc test, *p < .05 and **p < .01.

To directly assess engagement of the death‐execution machinery, we performed immunoblot analyses of key effector molecules. Consistent with the secretory profile, NLK‐deficient cells exhibited reduced levels of NLRP3, Caspase‐1 p10, GSDMD‐N, and cleaved Caspase‐3/7, accompanied by a pronounced increase in phosphorylated MLKL (Figure 6A, B and F–L). The elevation of p‐MLKL coincided with increased CXCL10 release, supporting a necroptosis‐associated execution pattern.

These molecular changes were further corroborated at the cellular level. Immunofluorescence microscopy revealed attenuated signals for GSDMD‐N and cleaved Caspase‐3, alongside intensified p‐MLKL staining in NLK‐deficient BMDMs (Figure 6C–E and M). To obtain complementary morphological support, we performed TEM. NLK‐deficient cells more frequently displayed pronounced organelle vacuolisation, cytoplasmic rarefaction, and plasma membrane rupture—ultrastructural features compatible with a necroptotic cell death, which were interpreted in conjunction with molecular marker–based analyses rather than used as stand‐alone criteria (Figure 6E and M).

Overall, NLK deficiency shifts macrophage death execution away from Caspase‐8–dependent PANoptotic signalling towards a necroptosis‐dominant phenotype, accompanied by corresponding alterations in inflammatory mediator release and executioner activation.

### 3.7 NLK deficiency shifts PANoptosome‐associated signalling towards RIPK1/3‐dependent necrosome activation

**FIGURE 7:**
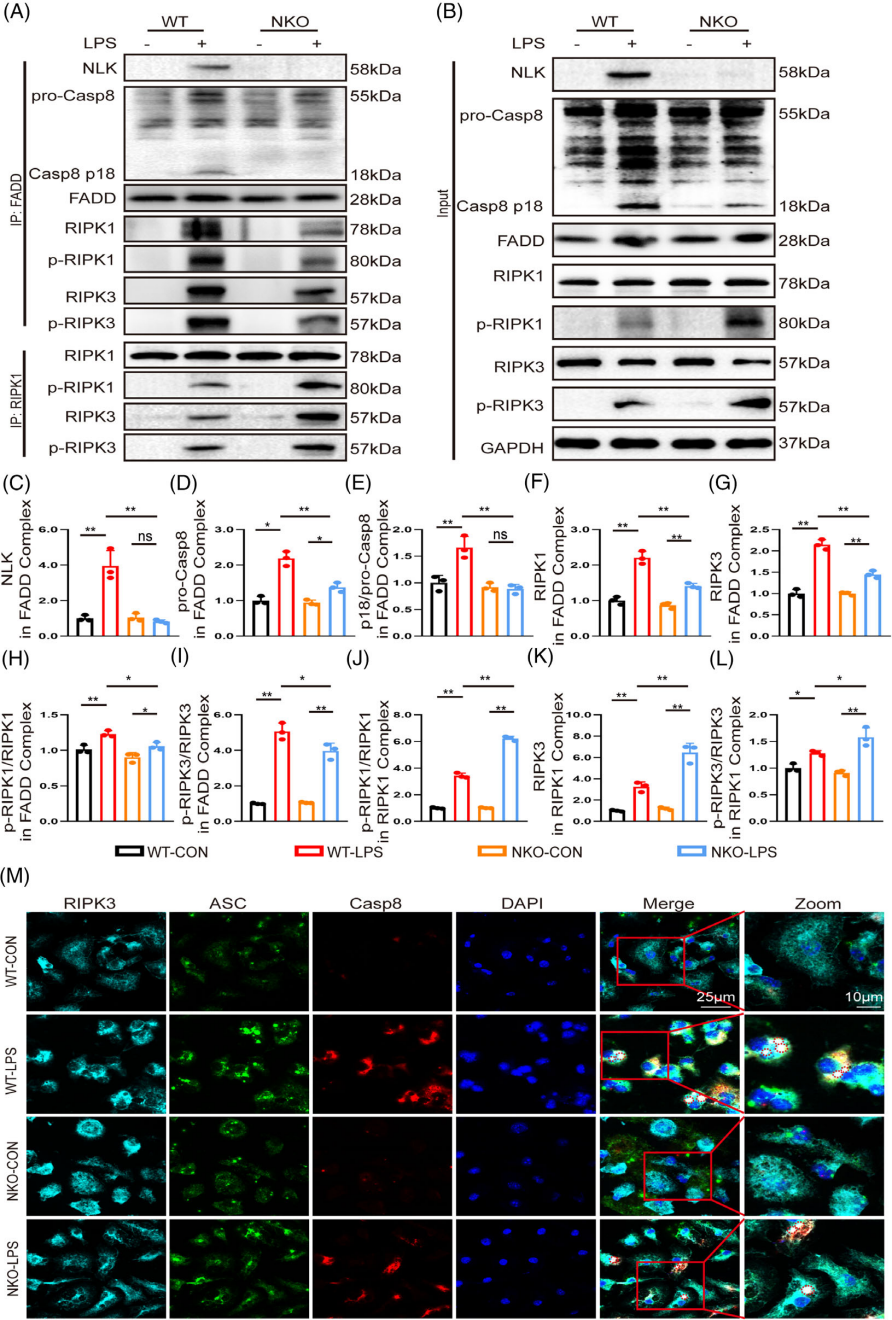
NLK deficiency impairs PANoptosome assembly and enhances RIPK1/3‐dependent necrosome formation in macrophages. (A, B) Representative immunoblots of FADD‐ and RIPK1‐associated complexes, together with corresponding input samples, in macrophages isolated from WT and NKO mice at 3 h post‐LPS. Co‐IP were probed for NLK, Caspase‐8, FADD, RIPK1, p‐RIPK1, RIPK3, and p‐RIPK3. (C–I) Quantification analysis of Caspase‐8, FADD, RIPK1, p‐RIPK1, RIPK3, and p‐RIPK3 in FADD‐associated complexes (*n* = 3 independent biological replicates). (J–L) Quantification analysis of p‐RIPK1, RIPK3, and p‐RIPK3 in RIPK1‐associated complexes (*n* = 3 independent biological replicates). (M) Representative confocal immunofluorescence images showing the co‑localisation of RIPK3 (cyan), ASC (green), and Caspase‑8 (red) in PBS‐ or LPS‐treated BMDMs. Merged images indicate RIPK3–ASC–Caspase‐8 colocalisation; boxed regions show magnified views. Scale bars: 25 µm (merged), 10 µm (zoomed). Statistical differences were analysed by one‑way ANOVA with Bonferroni's post hoc test, *p < .05 and **p < .01.

Cell viability analysis using PI/Calcein AM staining revealed that NLK‐deficient macrophages were more resistant to LPS‐induced cytotoxicity compared to WT controls, which is consistent with impaired PANoptosis‐associated signalling in these cells (Figure S2M and N). Consistent with the in vivo findings from the CLP model, LPS stimulation in WT BMDMs recapitulated the coordinated activation of PANoptotic signalling, characterised by robust Caspase‐8 cleavage and modest phosphorylation of RIPK1 and RIPK3 (Figures 7B and S2O–Q). In contrast, NLK deficiency dampened Caspase‐8 activation while markedly amplifying RIPK1/3 phosphorylation. Although LPS stimulation increased the expression of FADD, its abundance was comparable between WT and NKO macrophages (Figures 7B and S2R). Co‐IP with FADD further demonstrated that LPS stimulation promoted the recruitment of Caspase‐8 and efficient activation of PANoptosome‐associated signalling in WT macrophages, as evidenced by the recruitment of NLK, pro‐Caspase‐8, RIPK1, and RIPK3, together with enhanced Caspase‐8 cleavage and RIPK1/3 phosphorylation (Figure 7A and C–I). Conversely, in NLK‐deficient macrophages, recruitment of pro‐Caspase‐8 into FADD immunoprecipitates was reduced but not abolished, whereas Caspase‐8 activation was markedly impaired, as indicated by diminished p18 generation. This defect was accompanied by altered RIPK1 and RIPK3 phosphorylation, indicating dysfunctional PANoptosome signalling rather than a complete failure of complex assembly. Meanwhile, RIPK1 immunoprecipitates from NLK‐deficient macrophages showed increased RIPK1–RIPK3 complex formation together with higher levels of RIPK1 and RIPK3 phosphorylation, consistent with enhanced necrosome assembly and activation of necroptotic signalling (Figure 7A and J–L). Consistent with these findings, multicolour confocal microscopy demonstrated distinct ASC–Caspase‐8–RIPK3 cytoplasmic puncta in WT macrophages upon LPS stimulation, whereas these puncta were markedly fewer and less intense in NLK‐deficient cells (Figures 7M and S1Y), further supporting impaired PANoptosome‐associated signalling platform engagement in NLK‐deficient macrophages.

Collectively, these results indicate that NLK facilitates efficient Caspase‐8 activation within PANoptosome‐associated complexes while restraining RIPK1/3‐driven necrosome formation. Loss of NLK disrupts this balance, shifting macrophage cell death from PANoptosis towards necroptosis under LPS stimulation.

### 3.8 NLK interacts with the DED domain of Caspase‐8 to facilitate its activation

**FIGURE 8:**
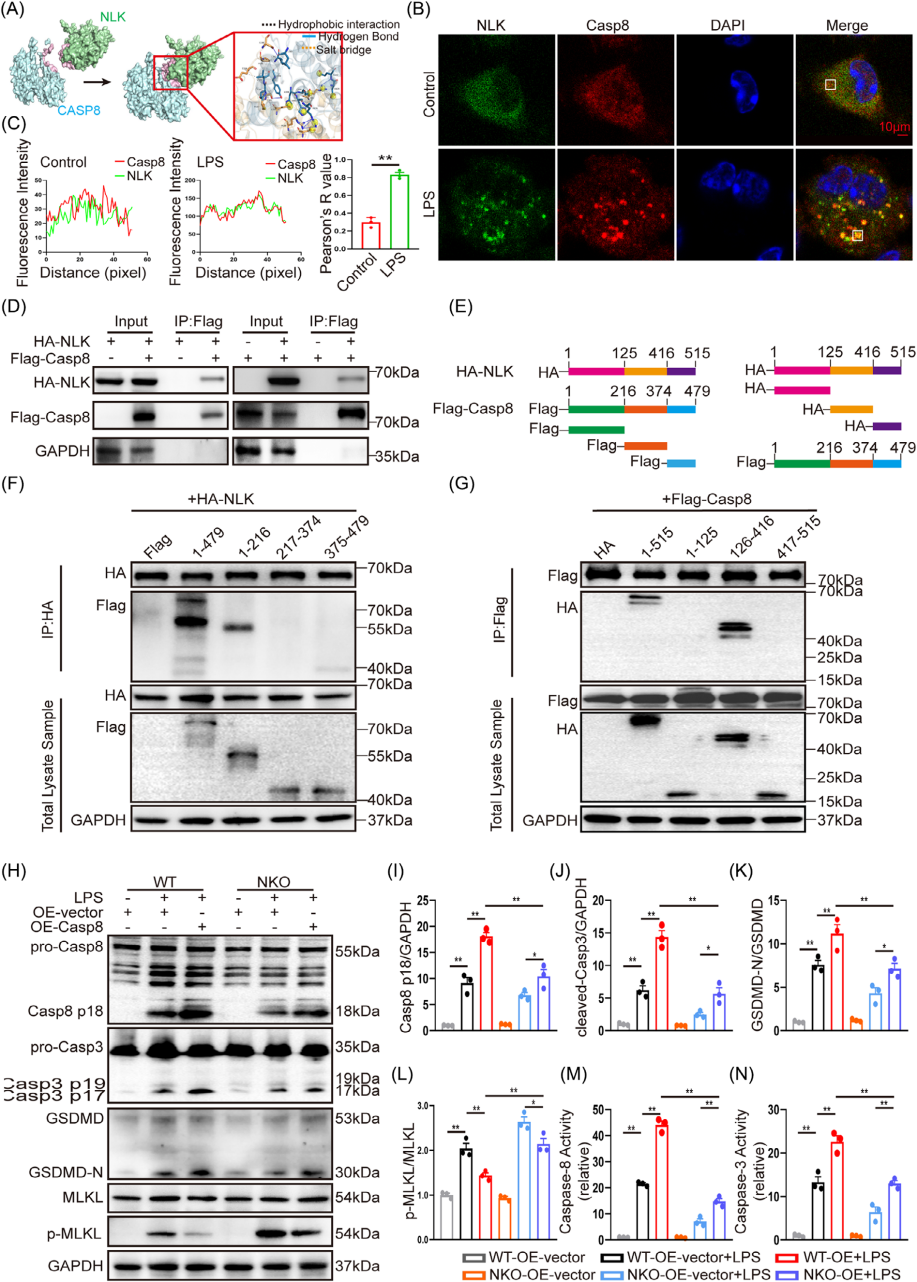
NLK interacts with the DED domain of Caspase‐8 to facilitate its activation. (A) Molecular docking model of the NLK–Caspase‑8 interface; inset depicts predicted hydrophobic contacts, hydrogen bonds and salt bridges. (B) Representative confocal immunofluorescence showing the subcellular localisation of NLK (green) and Caspase‑8 (red) in untreated BMDMs and after LPS stimulation; nuclei stained with DAPI (blue). Scale bars, 10 µm. (C) Quantitative analysis of fluorescence intensity and NLK–Caspase‑8 co‑localisation using Pearson's correlation coefficient (*n* = 3 independent biological replicates). (D) Co‑IP of HA‑tagged NLK and Flag‑tagged Caspase‑8 following transient co‑transfection in HEK293T cells (*n* = 3 independent biological replicates). (E) Schematic diagrams of Flag‐tagged Caspase‐8 truncation mutants (1–479, 1–216, 217–374, and 375–479) and HA‐tagged NLK truncation mutants (1–515, 1–125, 126–416, and 417–515) that were used for interaction mapping. (F) Representative Co‑IP results showing that HA‑NLK interacts with the full‐length Caspase‐8 and its N‐terminal fragment (residues 1–216) Representative blots from *n* = 3 independent biological replicates are shown. (G) Reciprocal Co‑IP demonstrated that Flag‑Caspase‑8 associated with NLK fragments containing residues 126–416. Representative blots from *n* = 3 independent biological replicates are shown. (H–L) Representative immunoblots and quantification of Caspase‐8, GSDMD, Caspase‐3, and p‐MLKL/MLKL levels in rescue experiments involving Caspase‐8 overexpression (*n* = 3 independent biological replicates). (M, N) Enzymatic activity assays of Caspase‐8 and Caspase‐3 in rescue experiments involving Caspase‐8 overexpression (*n* = 3 independent biological replicates). Statistical significance was assessed by the unpaired *t*‐test or two‑way ANOVA with Bonferroni's post hoc test; *p < .05 and **p < .01.

Based on these findings, we next examined whether NLK influences Caspase‐8, a central node within PANoptotic signalling. Molecular docking predicted a potential high‐affinity interface between NLK and Caspase‑8 (docking score −227.46; confidence score  .8248), mediated by hydrophobic and hydrogen‐bond interactions (Figure 8A). Consistent with this prediction, immunofluorescence in primary BMDMs revealed that NLK and Caspase‐8 displayed minimal colocalisation under basal conditions but formed distinct colocalised puncta upon LPS stimulation, reflected by increased Pearson's correlation coefficients (Figure 8B and C). Endogenous FADD‐IP assays confirmed that NLK was recruited together with Caspase‐8 into PANoptosome complexes under CLP and LPS stimulation (Figures 4A and 7A). In addition, heterologous Co‐IP in HEK293T cells (HA‐NLK + Flag‐Caspase‐8) also demonstrated an association between the two proteins under overexpression conditions (Figure 8D). Domain‐mapping with truncation mutants further localised the interaction to the N‐terminal death effector domains (DEDs, residues 1–216) of Caspase‑8, which mediate homotypic DED–DED interactions with adaptor proteins such as FADD, thereby enabling proximity‐induced dimerisation and autocleavage within PANoptosome assemblies.^31^, ^32^, ^33^ Reciprocally, Caspase‐8 associated with the central domain of NLK (residues 126–416) (Figure 8E–G).

Given that NLK deficiency impaired Caspase‐8 activation, we next asked whether reconstituting Caspase‐8 could functionally rescue NLK‐loss phenotypes. In WT macrophages, Caspase‐8 overexpression enhanced the generation of GSDMD‐N and cleaved Caspase‐3 upon LPS stimulation, accompanied by reduced p‐MLKL levels. Importantly, in NLK‐deficient macrophages, Caspase‐8 overexpression partially restored GSDMD‐N and cleaved Caspase‐3 expression and moderately suppressed MLKL phosphorylation, indicating incomplete restoration of pyroptotic and apoptotic activation as well as limited inhibition of necroptosis (Figure 8H–L). Enzymatic activity assays corroborated the immunoblotting data. LPS stimulation significantly increased Caspase‑8 and Caspase‑3 activities in WT macrophages, an effect further amplified by Caspase‑8 overexpression. In contrast, NLK‐deficient macrophages showed attenuated Caspase‐8 and Caspase‐3 activation upon LPS challenge. While Caspase‑8 reconstitution in NLK‑deficient cells partially restored Caspase‑8 activity and moderately increased Caspase‑3 activity, both remained significantly lower than those observed in Caspase‑8‑overexpressing WT cells under LPS stimulation (Figure 8M and N). Taken together, these data demonstrate that NLK engages Caspase‐8 through its DED domain and promotes efficient Caspase‐8 activation under inflammatory stimulation.

## 4 DISCUSSION

PANoptosis, an integrated form of cell death comprising pyroptosis, apoptosis, and necroptosis, plays a pivotal role in orchestrating immune responses during infection and sepsis. Within this network, Caspase‐8 functions as a pivotal molecular checkpoint that promotes apoptotic and pyroptotic execution while simultaneously restraining RIPK1/RIPK3‐dependent necroptosis. Although the downstream architecture of PANoptotic signalling has been extensively characterised, the upstream regulators that fine‐tune Caspase‐8 activation efficiency and bias death‐pathway selection remain incompletely understood. In this study, we identify Nemo‐like kinase (NLK) as a previously unrecognised regulator of Caspase‐8–mediated PANoptotic signalling, thereby linking inflammatory cues to macrophage fate decisions and systemic outcomes in sepsis.

To understand the mechanistic basis of this regulation, we next addressed how NLK engages Caspase‐8 at the molecular level. NLK is a serine/threonine kinase increasingly recognised for non‐catalytic scaffolding functions. Here, we extend this paradigm by demonstrating that NLK associates with Caspase‐8 through a central non‐catalytic region (residues 126–416), engaging the death effector domains (DEDs) that govern Caspase‐8 recruitment and proximity‐induced dimerisation within FADD–RIPK1/3–containing complexes. This association positions NLK at a critical structural interface required for efficient Caspase‐8 activation. Our data support a model in which NLK functions as an efficiency optimiser, facilitating Caspase‐8 incorporation into assembling PANoptosomes rather than nucleating complex assembly de novo. Consistent with this model, rescue experiments confirmed that NLK is required for optimal Caspase‐8 enzymatic activity and downstream PANoptotic execution. Thus, NLK promotes Caspase‐8 autocleavage primarily by enhancing recruitment and dimerisation efficiency, rather than through direct proteolytic or phospho‐regulatory mechanisms.

Consistent with this interpretation, NLK deficiency impaired pro–Caspase‐8 recruitment and autocleavage without abolishing the formation of death‐signalling scaffolds. As Caspase‐8 activation within these complexes is driven by DED‐mediated proximity, reduced recruitment efficiency translated into diminished Caspase‐8 activity. This attenuation was accompanied by enhanced RIPK1–RIPK3 interaction and increased MLKL phosphorylation, reflecting enhanced necrosome assembly and necroptotic execution. Importantly, the augmented RIPK1–RIPK3 interaction observed in NLK‐deficient macrophages should not be interpreted as evidence of direct NLK‐mediated regulation of necrosome components. Rather, it is best understood as a downstream consequence of impaired Caspase‐8–dependent suppression, whereby attenuation of apoptotic and pyroptotic checkpoints redistributes death signalling towards the necroptotic axis. These findings highlight that PANoptosome platform formation and Caspase‐8 activation, while functionally coupled, are mechanistically separable processes, with NLK acting preferentially at the level of Caspase‐8 engagement and activation efficiency.

PANoptotic signalling operates within a multilayered regulatory framework. Upstream sensors such as ZBP1 and the AIM2 inflammasome can nucleate PANoptosome formation, while core scaffold proteins including RIPK1 orchestrate recruitment of downstream effectors through defined interaction domains.^11^ Multiple checkpoints—including cFLIP‐mediated inhibition of Caspase‐8 and ubiquitin‐editing machineries such as LUBAC, cIAPs, and CYLD—further tune signalling outcomes.^34^, ^35^, ^36^ Within this complex hierarchy, NLK occupies a distinct regulatory niche. Unlike initiators or master scaffolds, NLK does not determine whether PANoptosis is triggered, but rather modulates how efficiently Caspase‐8 is activated once signalling platforms are formed. This positioning provides a mechanistic explanation for the moderate yet biologically meaningful phenotype observed upon NLK loss and suggests that NLK links inflammatory stimuli, such as TLR4 activation during Gram‐negative sepsis, to the quantitative execution of PANoptosis rather than its initiation. This fine‐tuning of death signalling, in turn, dictates the systemic inflammatory outcome. How NLK's recruitment‐optimising function is coordinated with these established PANoptotic regulators, and whether NLK intersects with upstream sensing or checkpoint mechanisms, remains an important question for future investigation.

Functionally, this molecular reprogramming translated into strikingly different inflammatory and pathological outcomes in vivo. In wild‐type mice, CLP‐induced sepsis triggered concurrent activation of pyroptosis, apoptosis, and necroptosis in macrophages, resulting in a mixed PANoptotic program associated with robust release of IL‐1β, IL‐18, IL‐6, and TNF‐α, culminating in cytokine storm, multiorgan injury, and high mortality. By contrast, NLK deficiency selectively attenuated the pyroptotic and apoptotic arms while enhancing necroptotic signalling. This death‐mode redistribution produced a distinct inflammatory profile characterised by reduced secretion of pyroptosis‐associated cytokines and broader pro‐inflammatory mediators, alongside elevated release of the necroptosis‐associated chemokine CXCL10.

Importantly, the protective phenotype observed in NLK‐deficient mice cannot be attributed to global macrophage depletion or impaired recruitment. Quantitative analyses demonstrated that total CD68^+^ macrophage abundance increased comparably in wild‐type and NLK‐deficient lungs following CLP. Instead, NLK loss reprogrammed the qualitative nature of the macrophage response, shifting functional polarisation from a predominantly CD68^+^CD86^+^ (M1‐like) towards a CD68^+^CD163^+^ (M2‐like) phenotype. This redistribution suggests that NLK deficiency alters macrophage behaviour and inflammatory output rather than suppressing macrophage accumulation, thereby contributing to a more regulated immune environment within septic lungs.

Beyond cell‐intrinsic death decisions, these findings have important implications for intercellular communication during sepsis. The shift from a mixed PANoptotic profile towards necroptosis in NLK‐deficient macrophages is predicted to alter the inflammatory signals transmitted to surrounding immune, stromal, and parenchymal cells. Reduced release of potent cytokines such as IL‐1β, IL‐18, IL‐6, and TNF‐α likely dampens paracrine feed‐forward inflammatory amplification that characterises cytokine storm. Conversely, increased production of CXCL10 suggests a qualitative change in inflammatory output, favouring chemokine‐driven immune cell recruitment over widespread cytokine dissemination. In parallel, the skewing of macrophage polarisation towards an M2‐like state may promote reparative cross‐talk with tissue compartments, supporting resolution of inflammation rather than its perpetuation. Together, these changes define a reprogrammed communication landscape that links NLK‐dependent control of macrophage death pathways to systemic protection in sepsis.

The role of necroptosis in sepsis is inherently context‐dependent.^37^, ^38^, ^39^ Although necroptosis is a lytic form of cell death that can exacerbate tissue injury under certain conditions, our data demonstrate a net protective effect when necroptosis replaces a more destructive mixed PANoptotic program. This underscores the principle that pathological outcomes are dictated not simply by whether cells die, but by how death pathways are balanced and integrated within inflammatory signalling networks. In this context, NLK emerges as a critical molecular switch that determines whether inflammatory cell death drives catastrophic tissue damage or a more controlled immune response.

The clinical relevance of the NLK–Caspase‐8 axis is supported by transcriptomic analyses of human sepsis cohorts, which revealed consistent upregulation of NLK in circulating monocytes and strong associations with PANoptotic effector genes, including *CASP8*, *CASP3*, *MLKL*, and *NLRP3*. Elevated NLK expression correlated with poor survival, suggesting that NLK‐high patients may represent a ‘hyper‐PANoptotic’ sepsis subset. These observations raise the possibility that NLK could serve as both a prognostic biomarker and a therapeutic target for stratifying and modulating dysregulated inflammatory cell death in sepsis.

Several limitations of this study should be acknowledged. First, our mechanistic investigations were primarily performed in murine macrophages and validated in the cecal ligation and puncture (CLP) model. Although this model is widely regarded as clinically relevant, it does not fully recapitulate the biological heterogeneity and complexity of human sepsis. Accordingly, future studies incorporating additional experimental models and human immune cells will be required to determine the generalisability of NLK‐mediated regulation across diverse septic contexts. Second, although NLK is a serine/threonine kinase, whether its kinase activity directly contributes to PANoptosome regulation remains unresolved and warrants further investigation using biochemical and structural approaches. Third, the in vivo phenotypes observed in this study were interpreted in the context of NLK deficiency mediated by the Csf1r‐iCre system, which predominantly targets monocyte–macrophage lineages. We acknowledge that Csf1r‐iCre–driven recombination is not strictly macrophage‐specific and may also occur, to varying extents, in other myeloid populations, including dendritic cells and granulocytes. Accordingly, while our data support a major contribution of macrophage‐intrinsic NLK signalling to the observed phenotypes, potential contributions from other Csf1r‐expressing immune cell subsets involved in sepsis pathogenesis cannot be fully excluded and warrant further investigation. Fourth, although our data support a functional association between NLK and Caspase‐8, confirmation of a direct physical interaction will require future biophysical studies using purified proteins, such as in vitro pull‐down assays or SPR/BLI analyses. Finally, all in vivo experiments in this study were conducted exclusively in male mice to minimise biological variability associated with the estrous cycle and sex hormone fluctuations, which are known to influence immune responses and sepsis outcomes.^40^ Future studies incorporating both sexes will be necessary to evaluate potential sex‐dependent effects of NLK signalling in sepsis.

In summary, we identify NLK as a novel modulator of Caspase‐8–associated PANoptotic signalling in sepsis. By enhancing the efficiency of DED‐mediated Caspase‐8 recruitment and activation, NLK calibrates the balance among pyroptosis, apoptosis, and necroptosis in macrophages. Our work thus defines NLK not as a simple on/off switch for cell death, but as a critical rheostat that shapes the quality and intensity of the inflammatory response. Disruption of this axis reprograms inflammatory cell death, alters macrophage inflammatory output with potential consequences for intercellular signalling, mitigates cytokine storm, and improves survival. The NLK–Caspase‐8 interface therefore represents a promising target for therapeutic strategies aimed at fine‐tuning, rather than broadly suppressing, inflammatory cell death in sepsis.

## AUTHOR CONTRIBUTIONS

Yun Xia, Ren‐qi Yao, and Hui‐fan Liu conceived and designed the study. Yun Xia, Jing Zuo, Die Wu, and Hai‐long Gong performed experiments and collected samples. Shi‐qi Wan and Shi Liu contributed to plasmid construction. Xing Wang and Hui‐min Zhou conducted data analysis. Guo‐qing Jing and Delida Aidebaike performed bioinformatics analyses of bulk RNA‐seq and scRNA‐seq datasets. Peng‐yue Zhao, Ning Dong, and Yao Wu contributed to data interpretation. Xiao‐jing Wu, Yong‐ming Yao, and Xue‐min Song supervised the study and secured funding. Yun Xia drafted the manuscript. Ren‐qi Yao and Hui‐fan Liu critically revised it for important intellectual content. All authors read and approved the final version of the manuscript.

## CONFLICT OF INTEREST STATEMENT

The authors declare no competing interests.

## ETHICS STATEMENT

All animal procedures were approved by the Animal Ethics Committee of Wuhan University (Approval No. WP2022‐12118) and were conducted in accordance with the NIH Guide for the Care and Use of Laboratory Animals.

## Supporting information



Supporting Information

**Figure S1**. (A) UMAP visualisation of eight immune cell populations identified from PBMC single‐cell RNA‐seq data of patients with sepsis and healthy controls in the GSE167363 dataset (resolution  .1). (B) Spearman's correlation heatmap in monocytes showing positive associations between NLK and PANoptosis‐related genes. (C–F) Analysis of macrophage infiltration and polarisation in lung tissue following CLP‐induced sepsis. (C) Representative immunofluorescence images staining for CD68 with CD86 or CD163. (D) Quantification of total CD68^+^ macrophage abundance. Data are presented as the fold change relative to the mean value of the WT‐Sham group, which was set to 1. (E, F) Quantification of CD68^+^CD86^+^ (E) and CD68^+^CD163^+^ (F) macrophage subsets, expressed as the percentage of CD68^+^ macrophages within each field. Scale bar: 100 µm; *n* = 6 mice per group. (G–J) Quantification of Caspase‐8, FADD, RIPK1, p‐RIPK1, RIPK3, and p‐RIPK3 levels in input lysates from sham‐ or CLP‐treated WT and NLK‐deficient mice (*n* = 3 independent biological replicates). (K) Quantification of immunofluorescence colocalisation of lung macrophages stained for CD68 (green), Caspase‑8 (red), and ASC (cyan) (*n* = 3 independent biological replicates).

**Figure S2**. (A–G) Quantitative analysis of time‐course immunoblot data in Figure 5B and C, showing NLK and PANoptosis‐related markers, including apoptosis (cleaved Caspase‐3 and Caspase‐7), necroptosis (MLKL and p‐MLKL), and pyroptosis (NLRP3, pro‐/cleaved Caspase‐1, and full‐length/N‐terminal GSDMD), following LPS treatment in BMDMs (*n* = 3 independent biological replicates). (H–L) ELISA quantification of TNF‐α, IL‐6, IL‐1β, IL‐18, and CXCL10 levels from WT and NLK‐deficient BMDMs at 12 h post‐LPS stimulation (*n* = 3 independent biological replicates). (M, N) Representative PI/calcein AM staining and quantification of WT and NLK‑deficient BMDMs treated with PBS or LPS for 12 h (Scale bar: 25 µm, *n* = 3 independent biological replicates). (O–R) Quantification of Caspase‐8, FADD, RIPK1, p‐RIPK1, RIPK3, and p‐RIPK3 levels in input lysates from PBS‐ or LPS‐treated WT and NLK‐deficient BMDMs (*n* = 3 independent biological replicates). (S) Quantification of the co‑localisation of RIPK3 (cyan), ASC (green), and Caspase‑8 (red) in PBS‐ or LPS‐treated BMDMs (*n* = 3 independent biological replicates). Statistical differences were analysed by one‑way ANOVA with Bonferroni's post hoc test, *p < .05 and **p < .01.

Supporting Information

Supporting Information

Supporting Information

## Data Availability

All datasets generated or analysed during this study are included in the main manuscript and supplementary files.
