# Supplementary material for: NLK facilitates Caspase‐8 activation to drive macrophage PANoptosis in sepsis
Source: Clin Transl Med. 2026 Feb 11;16(2):e70616. doi: 10.1002/ctm2.70616 (PMC12894773; doi:10.1002/ctm2.70616)
Supplement: Supplementary file 1 — Supporting Information [file CTM2-16-e70616-s003.docx]

**Supplemental Table 1.** The evaluation criteria for organ pathological changes.

| **Organs** | **Scoring Criteria** |
| --- | --- |
| **Lung** | Pulmonary Edema |
|  | Alveolar/Interstitial Inflammation |
|  | Alveolar Hemorrhage |
|  | Atelectasis |
|  | Hyaline Membrane Formation. |
| **Liver** | Hepatocellular Vacuolization |
|  | Inflammatory Infiltration |
|  | Interstitial Leukocyte Infiltration |
| **Renal** | Tubular Dilatation |
|  | Epithelial Necrosis |
|  | Interstitial Leukocyte Infiltration |
| **Cardiac** | Myofibrillar Degeneration |
|  | Interstitial Edema |
|  | Perivascular Inflammatory Infiltration |

Histopathological lesions were graded on a 0–3 severity scale: 0=normal histoarchitecture; 1=focal/minimal changes (<25% involvement); 2=moderate lesions (25-50% involvement); 3=extensive alterations (>50% involvement). Higher cumulative scores reflected greater pathological severity across examined tissues.
