# Supplementary figures and images for: NLK facilitates Caspase‐8 activation to drive macrophage PANoptosis in sepsis
Source: Clin Transl Med. 2026 Feb 11;16(2):e70616. doi: 10.1002/ctm2.70616 (PMC12894773; doi:10.1002/ctm2.70616)

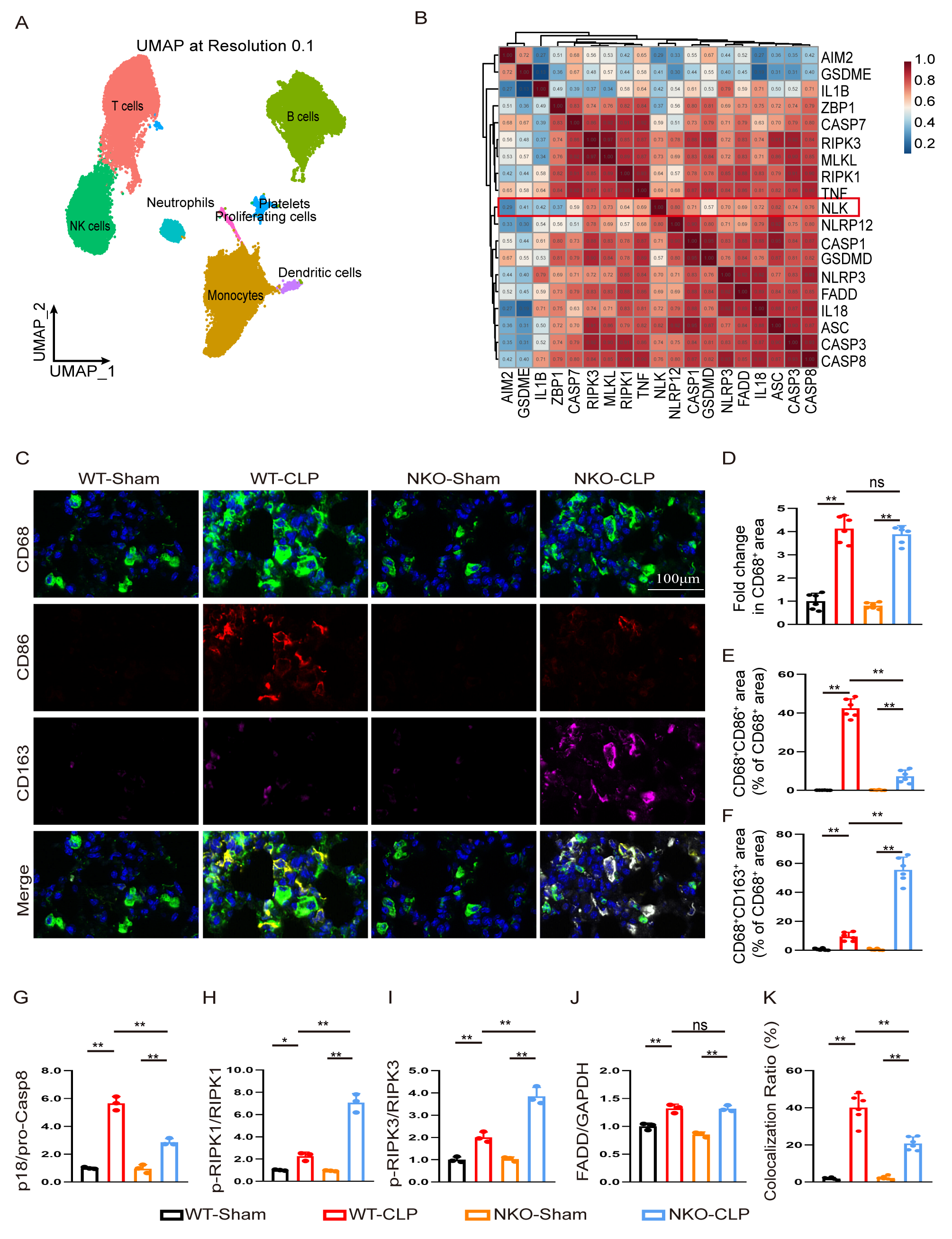

Supplement: Supplementary file 2 — Figure S1. (A) UMAP visualisation of eight immune cell populations identified from PBMC single‐cell RNA‐seq data of patients with sepsis and healthy controls in the GSE167363 dataset (resolution .1). (B) Spearman's correlation heatmap in monocytes showing positive associations between NLK and PANoptosis‐related genes. (C–F) Analysis of macrophage infiltration and polarisation in lung tissue following CLP‐induced sepsis. (C) Representative immunofluorescence images staining for CD68 with CD86 or CD163. (D) Quantification of total CD68+ macrophage abundance. Data are presented as the fold change relative to the mean value of the WT‐Sham group, which was set to 1. (E, F) Quantification of CD68+CD86+ (E) and CD68+CD163+ (F) macrophage subsets, expressed as the percentage of CD68+ macrophages within each field. Scale bar: 100 µm; n = 6 mice per group. (G–J) Quantification of Caspase‐8, FADD, RIPK1, p‐RIPK1, RIPK3, and p‐RIPK3 levels in input lysates from sham‐ or CLP‐treated WT and NLK‐deficient mice (n = 3 independent biological replicates). (K) Quantification of immunofluorescence colocalisation of lung macrophages stained for CD68 (green), Caspase‑8 (red), and ASC (cyan) (n = 3 independent biological replicates). [file CTM2-16-e70616-s002.tif]

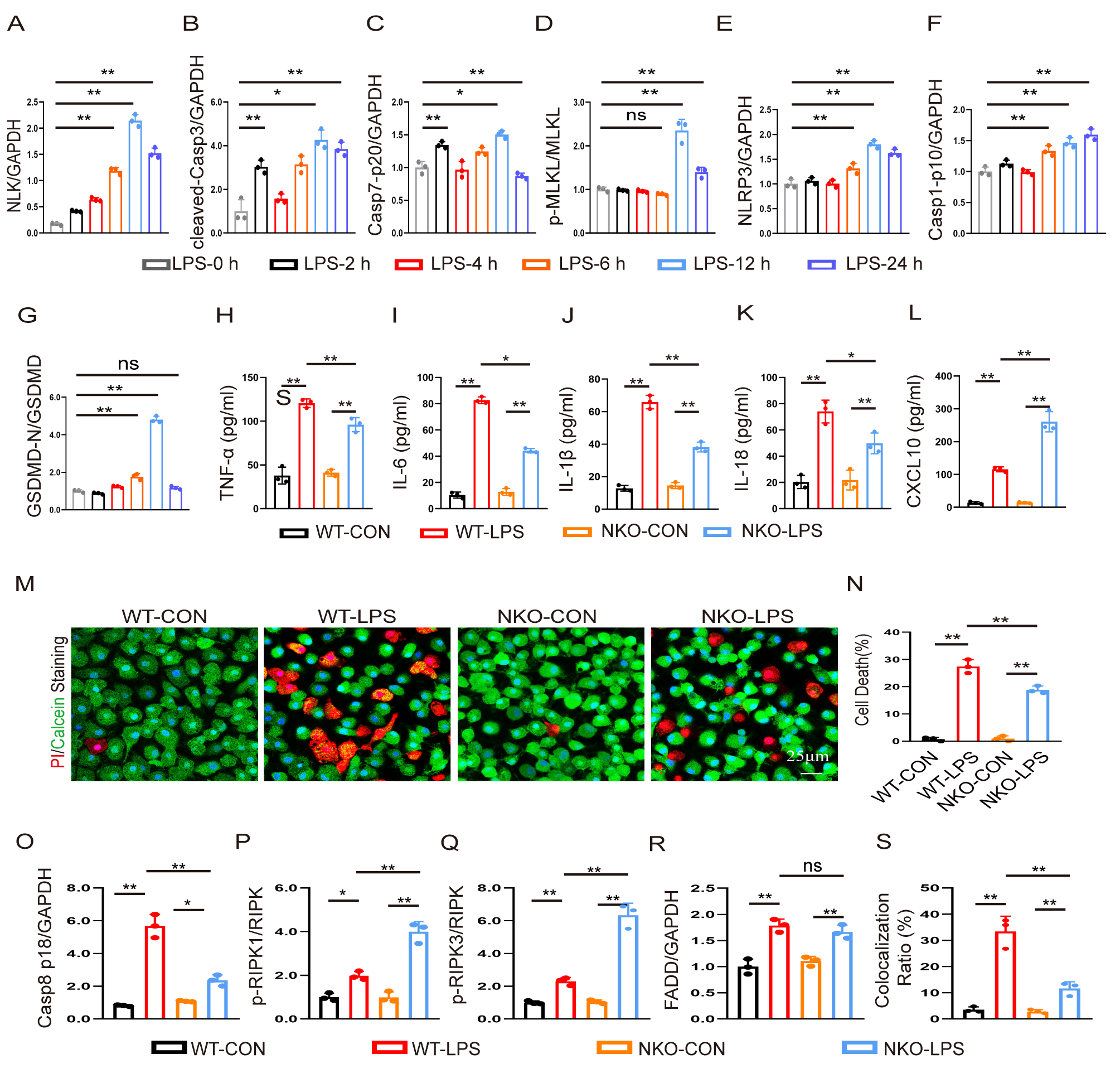

Supplement: Supplementary file 3 — Figure S2. (A–G) Quantitative analysis of time‐course immunoblot data in Figure 5B and C, showing NLK and PANoptosis‐related markers, including apoptosis (cleaved Caspase‐3 and Caspase‐7), necroptosis (MLKL and p‐MLKL), and pyroptosis (NLRP3, pro‐/cleaved Caspase‐1, and full‐length/N‐terminal GSDMD), following LPS treatment in BMDMs (n = 3 independent biological replicates). (H–L) ELISA quantification of TNF‐α, IL‐6, IL‐1β, IL‐18, and CXCL10 levels from WT and NLK‐deficient BMDMs at 12 h post‐LPS stimulation (n = 3 independent biological replicates). (M, N) Representative PI/calcein AM staining and quantification of WT and NLK‑deficient BMDMs treated with PBS or LPS for 12 h (Scale bar: 25 µm, n = 3 independent biological replicates). (O–R) Quantification of Caspase‐8, FADD, RIPK1, p‐RIPK1, RIPK3, and p‐RIPK3 levels in input lysates from PBS‐ or LPS‐treated WT and NLK‐deficient BMDMs (n = 3 independent biological replicates). (S) Quantification of the co‑localisation of RIPK3 (cyan), ASC (green), and Caspase‑8 (red) in PBS‐ or LPS‐treated BMDMs (n = 3 independent biological replicates). Statistical differences were analysed by one‑way ANOVA with Bonferroni's post hoc test, *p < .05 and **p < .01. [file CTM2-16-e70616-s001.tif]

# Annotated Cell Types

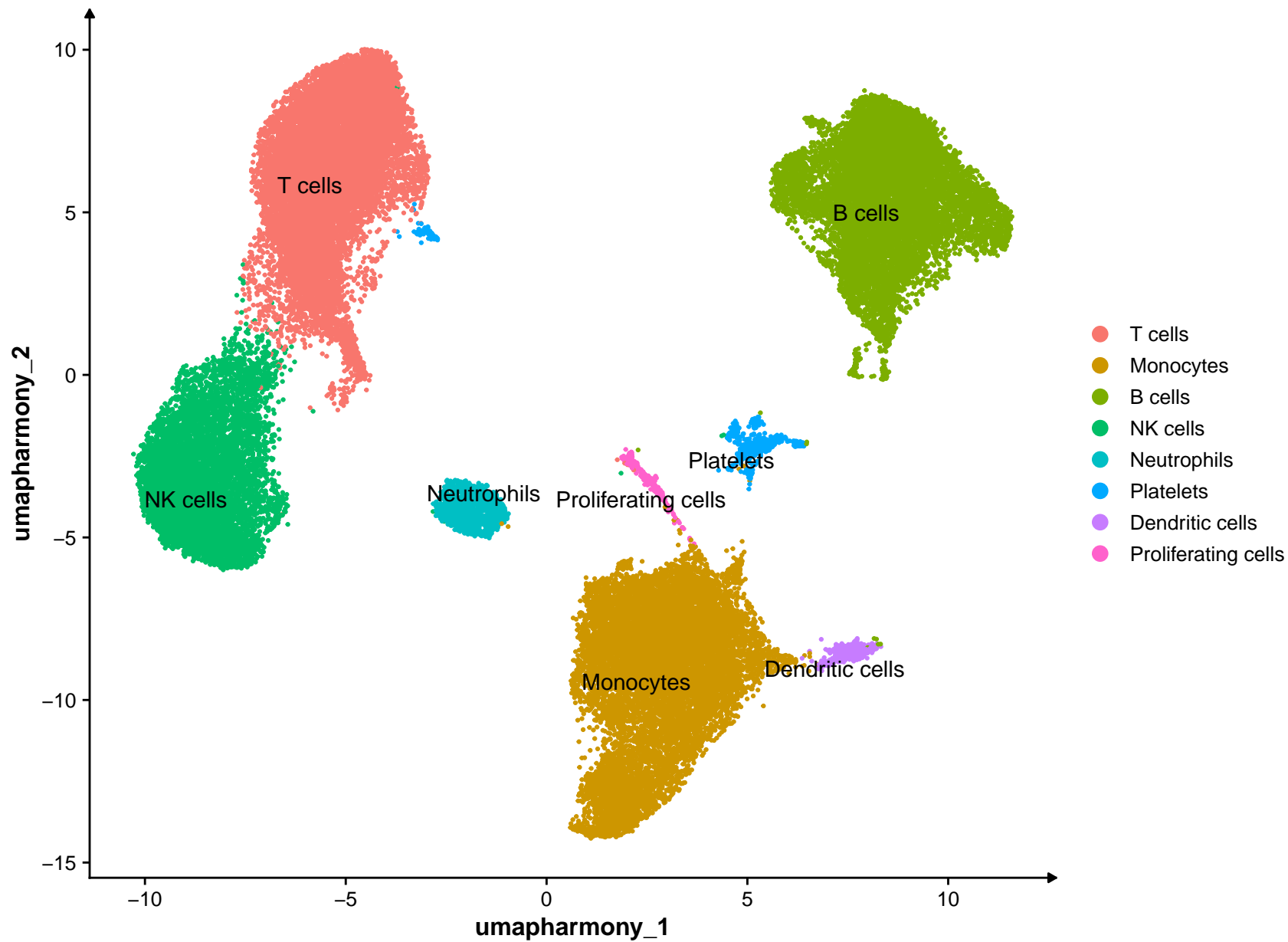

Supplement: Supplementary file 6 — Supporting Information [file CTM2-16-e70616-s004.zip › Supplementary_CellType_Annotation_Validation/annotated_umap.pdf]

# Cell Type Marker Expression

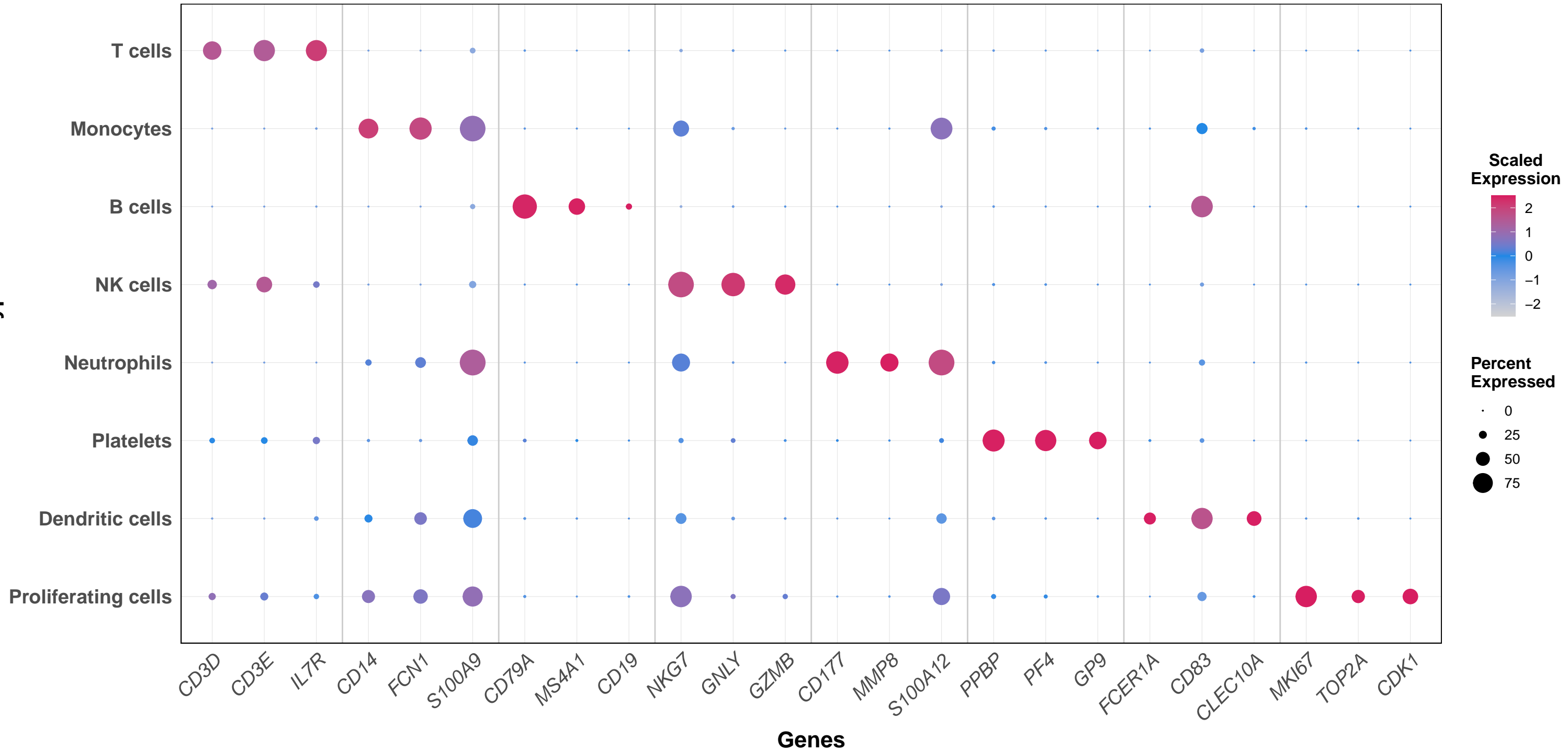

Supplement: Supplementary file 6 — Supporting Information [file CTM2-16-e70616-s004.zip › Supplementary_CellType_Annotation_Validation/marker_gene_dotplot_selected.pdf]

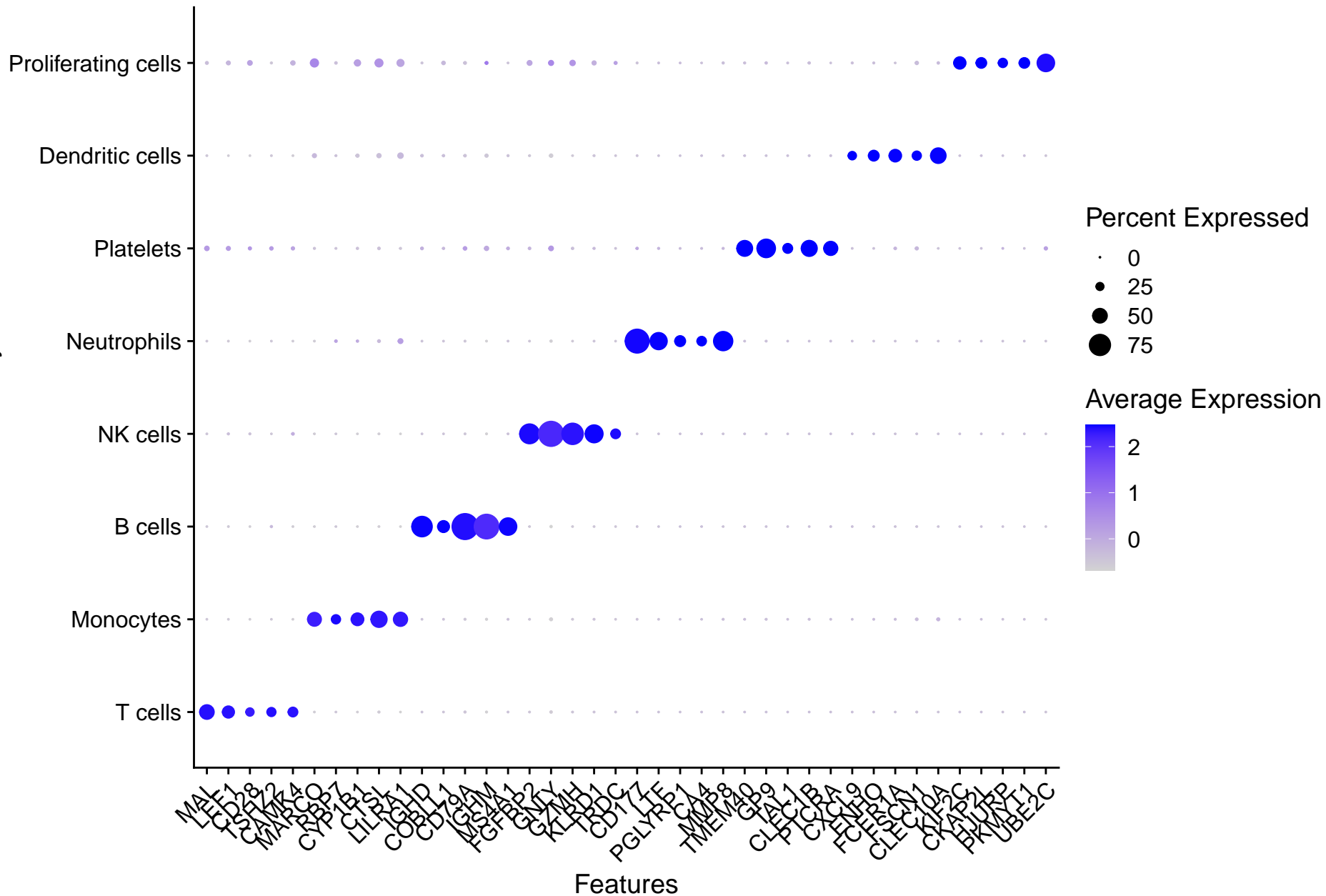

Supplement: Supplementary file 6 — Supporting Information [file CTM2-16-e70616-s004.zip › Supplementary_CellType_Annotation_Validation/top5_marker_bubble_plot.pdf]
