# Supplementary material for: NLK facilitates Caspase‐8 activation to drive macrophage PANoptosis in sepsis
Source: Clin Transl Med. 2026 Feb 11;16(2):e70616. doi: 10.1002/ctm2.70616 (PMC12894773; doi:10.1002/ctm2.70616)

NLK Csf1r-iCre

1. Genotyping strategy

Primers1:

F1: 5’-AGACATCAGCTGCCAAATAGCCA-3’

R1: 5’-TGTCTCTGAAGTCCCAGTGTTAGCTTG-3’

Homozygotes: one band with 375 bp

Heterozygotes: two bands with 375 bp and 270 bp

Wildtype allele: one band with 270 bp

Primers2:

iCre-MF: 5’-GGACTATGCTAACCTGCCAAGC-3’

iCre-MR: 5’-GGAATGCTCGTCAAGAAGACAG-3’

Product size: 350 bp

Primers3:

iCre-WF: 5’-GGACTATGCTAACCTGCCAAGC-3’

iCre-WR: 5’-CAGCTTACCCACAGCCTTTGAG-3’

Homozygotes: 538 bp

Wildtype allele: 2195 bp

2. PCR reaction

2.1 DNA Extraction

Genomic DNA was extracted from mouse tail biopsies using the TaKaRa MiniBEST Universal Genomic DNA Extraction Kit (Ver.5.0, Code No. 9765).

a. Add 180 μL of Buffer GL, 20 μL of Proteinase K and 10 μL of RNase A per tail piece (2-5 mm) in a microcentrifuge tube.

b. Incubate the tube at 56℃ overnight.

c. Spin in microcentrifuge at 12,000 rpm for 2 minutes to remove impurities.

d. Add 200 μL Buffer GB and 200 μL absolute ethyl alcohol with sufficient mixing.

e. Place the spin Column in a collection tube. Apply the sample to the spin and centrifuge at 12,000 rpm for 2 min. Discard flow-through.

f. Add 500 μL Buffer WA to the spin column and centrifuge at 12,000 rpm for 1 min. Discard flow-through.

g. Add 700 μL Buffer WB to the spin column and centrifuge at 12,000 rpm for 1 min. Discard flow-through.

h. Repeat step g.

i. Place the spin Column in a collection tube and centrifuge at 12,000 rpm for 2 min.

j. Place the spin Column in a new 1.5ml tube. Add 50~200 μL sterilized water or elution buffer to the center of the column membrane and let the column stand 5min.

k. To elute DNA, centrifuge the column at 12,000 rpm for 2 min. To increase the yield of DNA, add the flow-through and/or 50~200 μL sterilized water or elution buffer to the center of the spin column membrane and let the column stand 5 min. Centrifuge at 12,000 rpm for 2 min.

l. Quantify to genomic DNA. Eluted genomic DNA can be quantified by electrophoresis or electrophoresis.

2.2 PCR Mixture (primer concentration: 10μM):

| Component | x1 |
| --- | --- |
| ddH2O | 9.0 μl |
| Product primer F | 1.0 μl |
| Product primer R | 1.0 μl |
| Premix Taq | 12.5 μl |
| DNA | 1.5 μl |
| Total | 25 μl |

2.3 PCR Reaction Conditions:

| Step | Temp. | Time | Cycles |
| --- | --- | --- | --- |
| Initial denaturation | 94 °C | 3 min |  |
| Denaturation | 94 °C | 30 s | 35 x |
| Annealing | 60 °C | 35 s |  |
| Extension | 72 °C | 35 s |  |
| Additional extension | 72 °C | 5 min |  |

2.4 Relevant Reagents:

TrizmaHydrochloride Solution Sigma, Cat. No. T2663

2 × Taq Master Mix (Dye Plus) Vazyme, P222-C2

Agarose BIOWEST AGAROSE, REGULAR

DNA Marker Thermo Scientific GeneRuler 100 bp DNA Ladder #SM0242

Tris Bio Basic Inc, TBO194-500g

EDTA Shanghai Sangon, 0105-500g

Boric Acid, Shanghai Sangon, 0588-500g

3. Results

Primers1: (Homozygotes: one band with 375 bp)


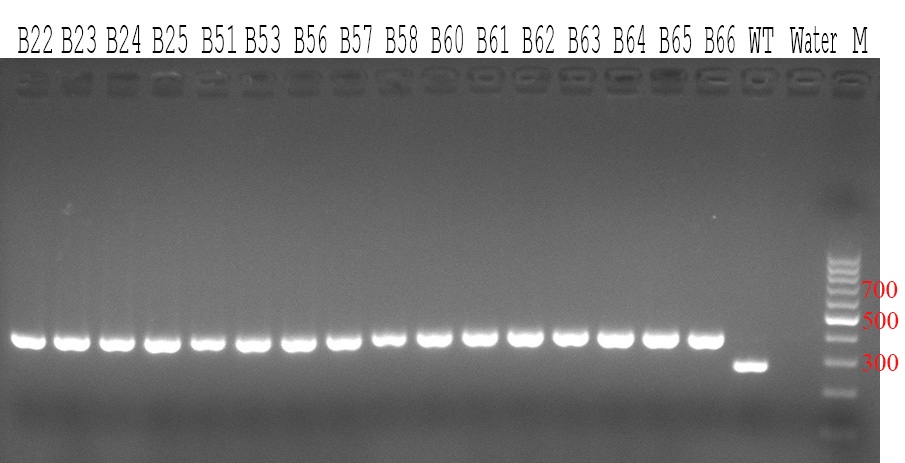


Primers2: (350 bp)


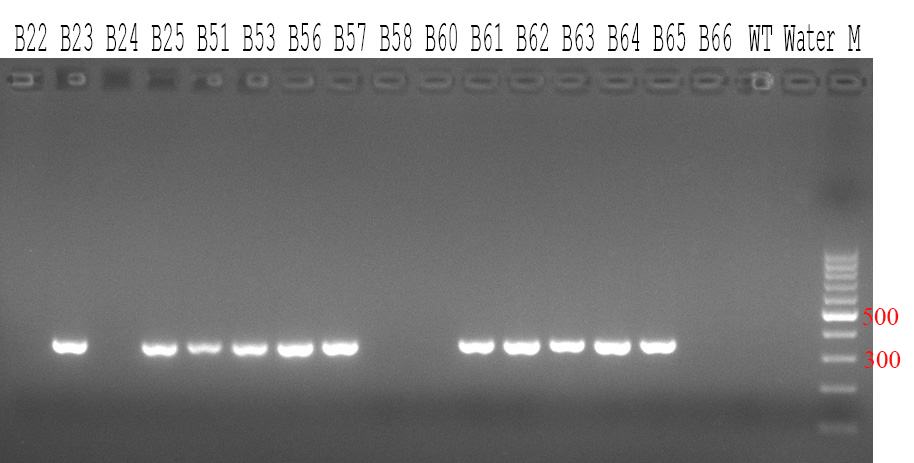


Primers3: (Homozygotes: 538 bp; Wildtype allele: 2195 bp)


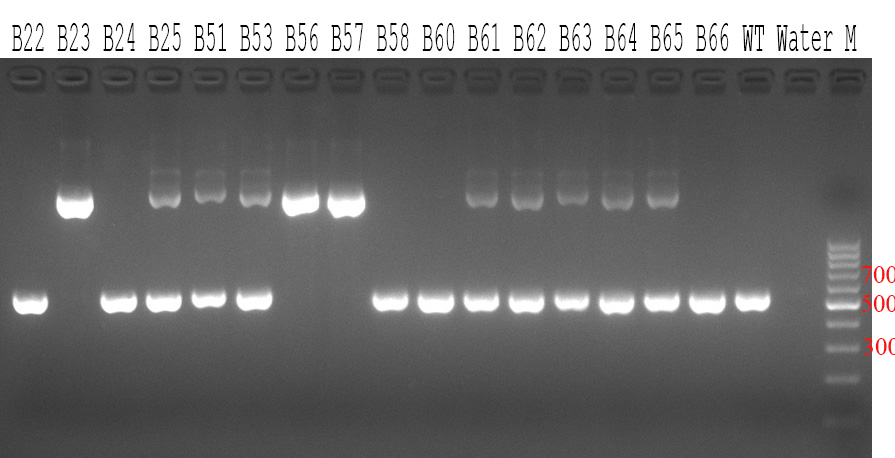


NLK^fl/fl/Csf1r-iCre^ (NKO): B25, B51, B53, B61, B62, B63, B64, B65

NLK^fl/fl^ (WT): B22, B24, B58, B60, B66

These results confirmed the successful generation of NLK^fl/fl/Csf1r-iCre^ mice used in subsequent experiments.

4. qPCR result analysis derived from raw data shown in Supplementary Table 2
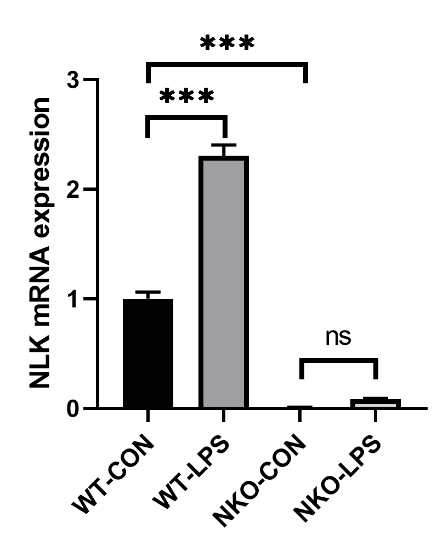

Supplement: Supplementary file 5 — Supporting Information [file CTM2-16-e70616-s005.docx]
