# Supplementary material for: NLK facilitates Caspase‐8 activation to drive macrophage PANoptosis in sepsis
Source: Clin Transl Med. 2026 Feb 11;16(2):e70616. doi: 10.1002/ctm2.70616 (PMC12894773; doi:10.1002/ctm2.70616)
Supplement: Supplementary file 6 — Supporting Information [file CTM2-16-e70616-s004.zip › Supplementary_CellType_Annotation_Validation/annotated_markers_heatmap.pdf]

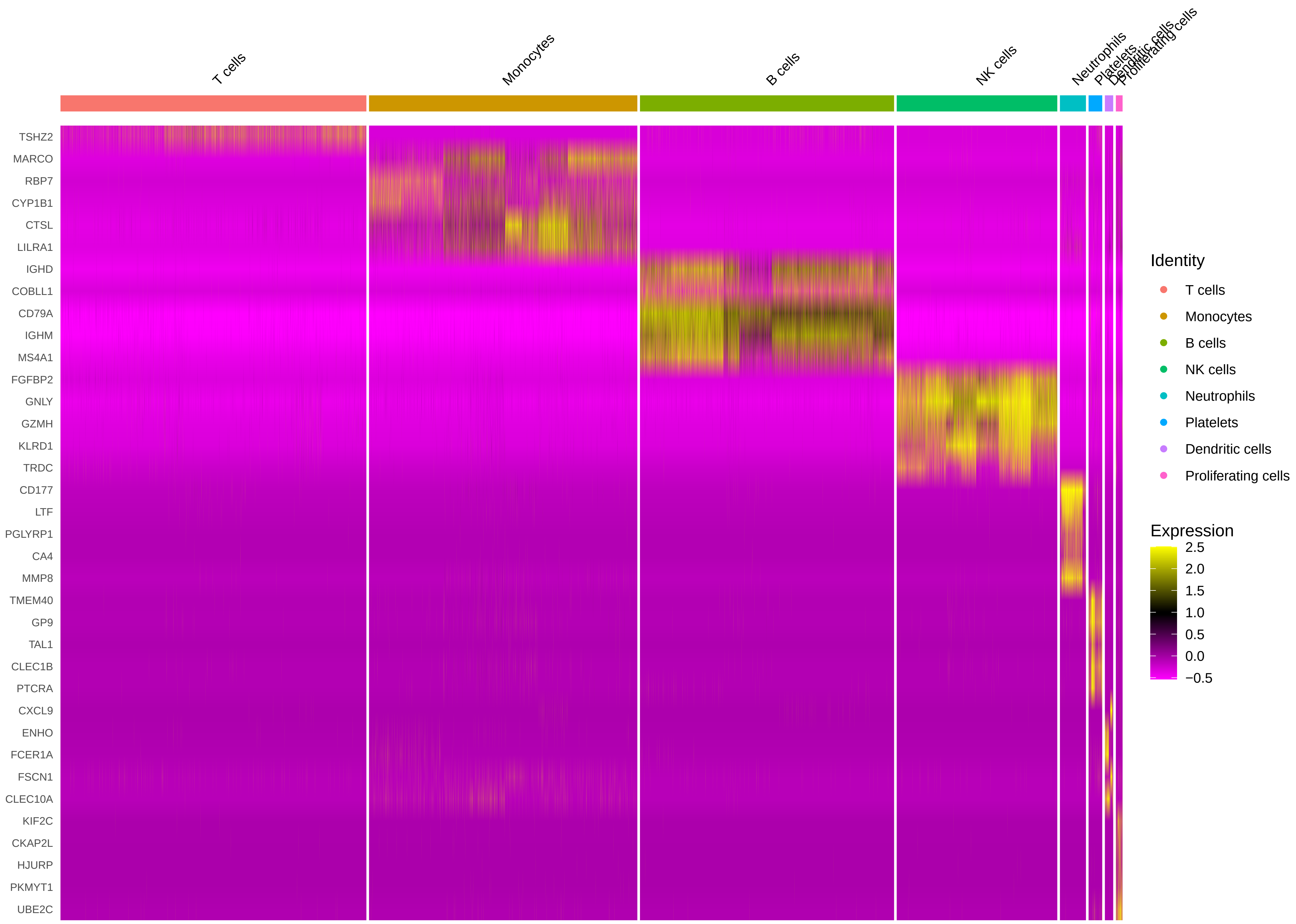
T cells
Monocytes
B cells
NK cells
Neutrophils
Platelets
Dendritic cells
Proliferating cells
TSHZ2
MARCO
RBP7
CYP1B1
CTSL
LILRA1
IGHD
COBLL1
CD79A
IGHM
MS4A1
FGFBP2
GNLY
GZMH
KLRD1
TRDC
CD177
LTF
PGLYRP1
CA4
MMP8
TMEM40
GP9
TAL1
CLEC1B
PTCRA
CXCL9
ENHO
FCER1A
FSCN1
CLEC10A
KIF2C
CKAP2L
HJURP
PKMYT1
UBE2C
Identity
T cells
Monocytes
B cells
NK cells
Neutrophils
Platelets
Dendritic cells
Proliferating cells
Expression
2.5
2.0
1.5
1.0
0.5
0.0
−0.5
