# Supplementary material for: NLK facilitates Caspase‐8 activation to drive macrophage PANoptosis in sepsis
Source: Clin Transl Med. 2026 Feb 11;16(2):e70616. doi: 10.1002/ctm2.70616 (PMC12894773; doi:10.1002/ctm2.70616)
Supplement: Supplementary file 6 — Supporting Information [file CTM2-16-e70616-s004.zip › Supplementary_CellType_Annotation_Validation/QC_comparison.pdf]

Quality Control Metrics for Single−cell RNA Sequencing

Total cells: Pre−QC = 61,695 | Post−QC = 49,628 (Retention: 80.4%)

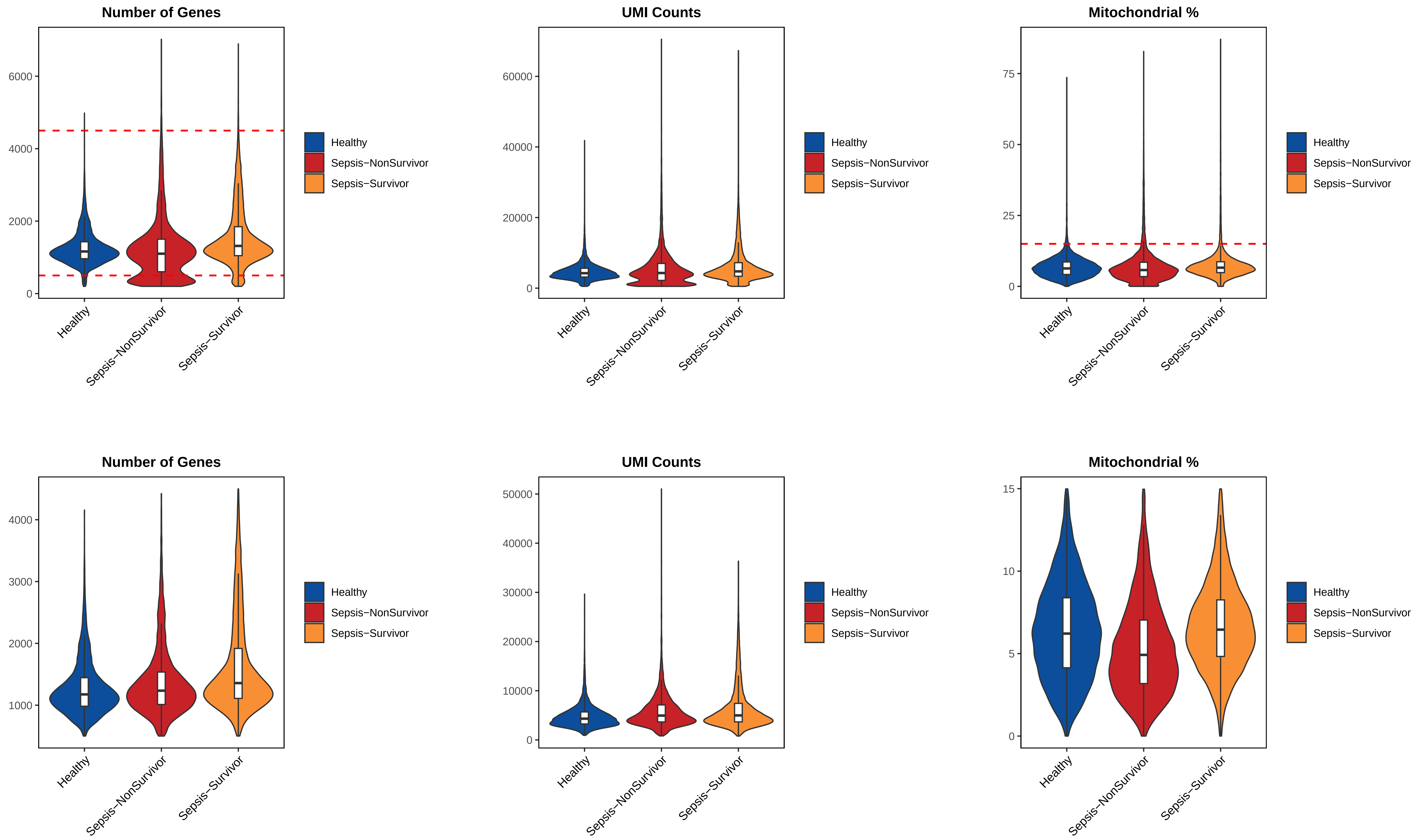

QC thresholds: Genes (500−4500), MT% <15%, HB% <1%
