# Supplementary material for: NLK facilitates Caspase‐8 activation to drive macrophage PANoptosis in sepsis
Source: Clin Transl Med. 2026 Feb 11;16(2):e70616. doi: 10.1002/ctm2.70616 (PMC12894773; doi:10.1002/ctm2.70616)
Supplement: Supplementary file 6 — Supporting Information [file CTM2-16-e70616-s004.zip › Supplementary_CellType_Annotation_Validation/Supplementary Methods Cell Annotation Validation.pdf]

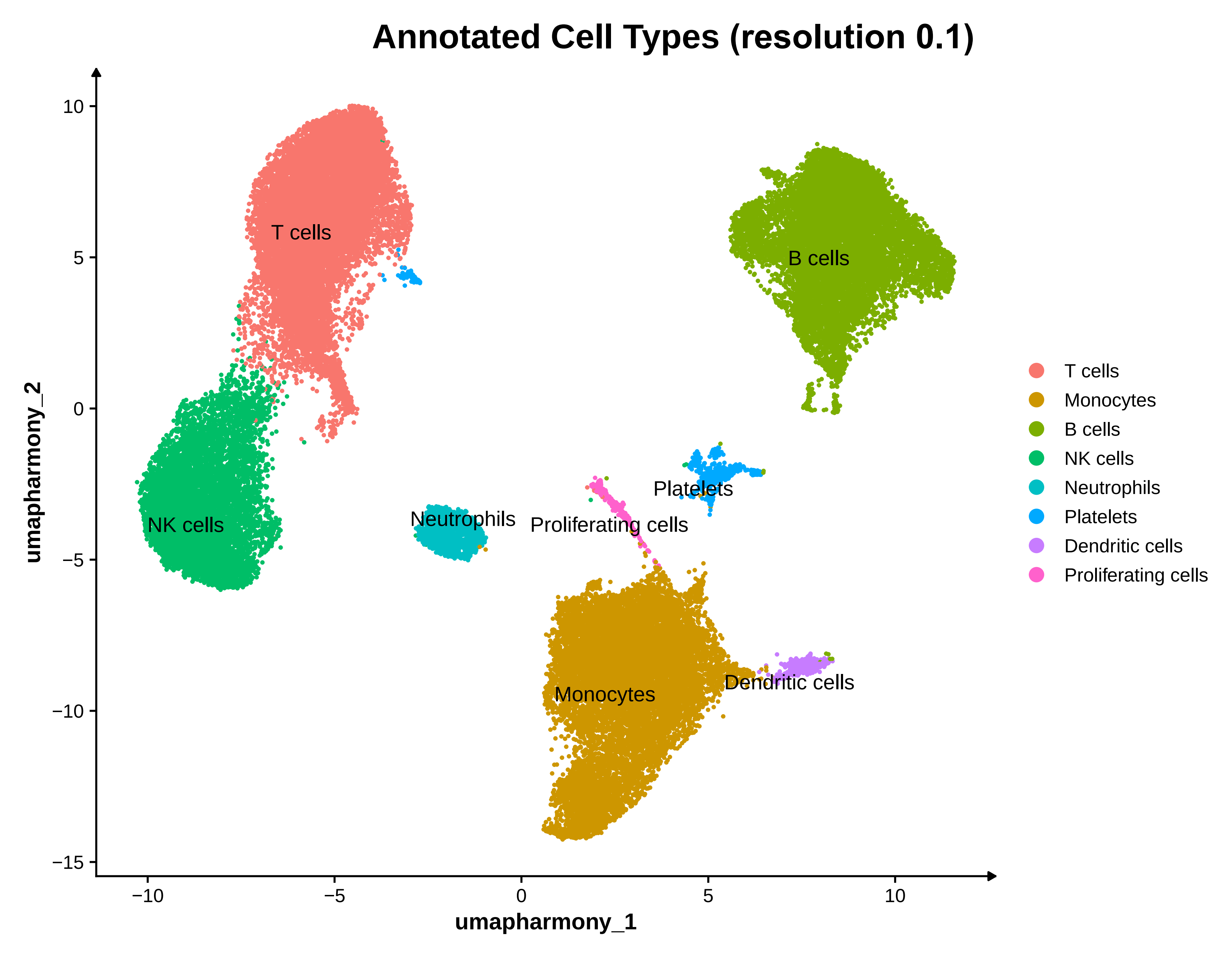

Annotated Cell Types (resolution 0.1)
umapharmony_2
umapharmony_1
10
5
0
−5
−10
−15
−10
−5
0
5
10
T cells
B cells
NK cells
Neutrophils
Proliferating cells
Platelets
Monocytes
Dendritic cells
T cells
Monocytes
B cells
NK cells
Neutrophils
Platelets
Dendritic cells
Proliferating cells

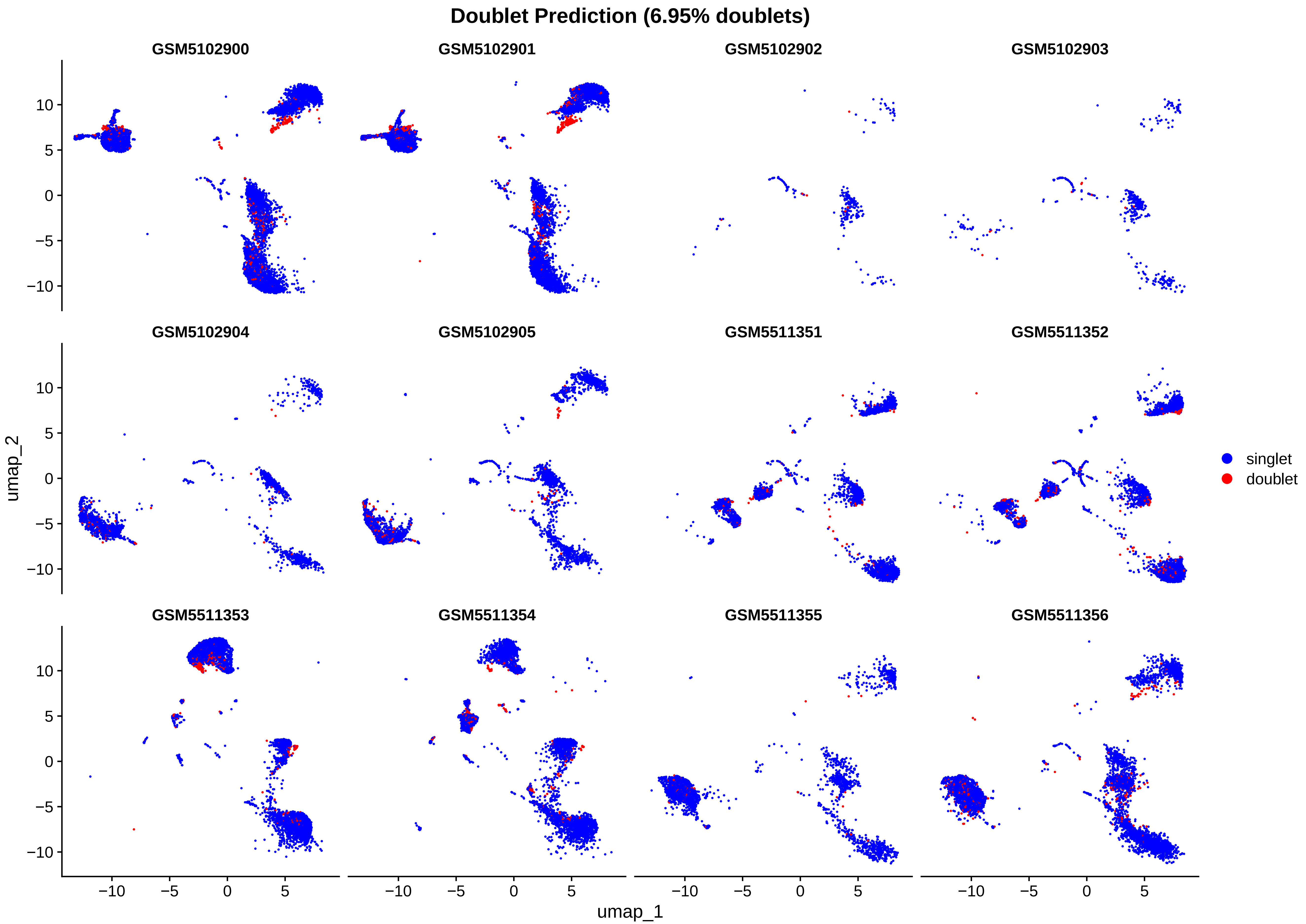

Doublet Prediction (6.95% doublets)
GSM5102900
GSM5102901
GSM5102902
GSM5102903
GSM5102904
GSM5102905
GSM5511351
GSM5511352
GSM5511353
GSM5511354
GSM5511355
GSM5511356
umap_2
umap_1
singlet
doublet

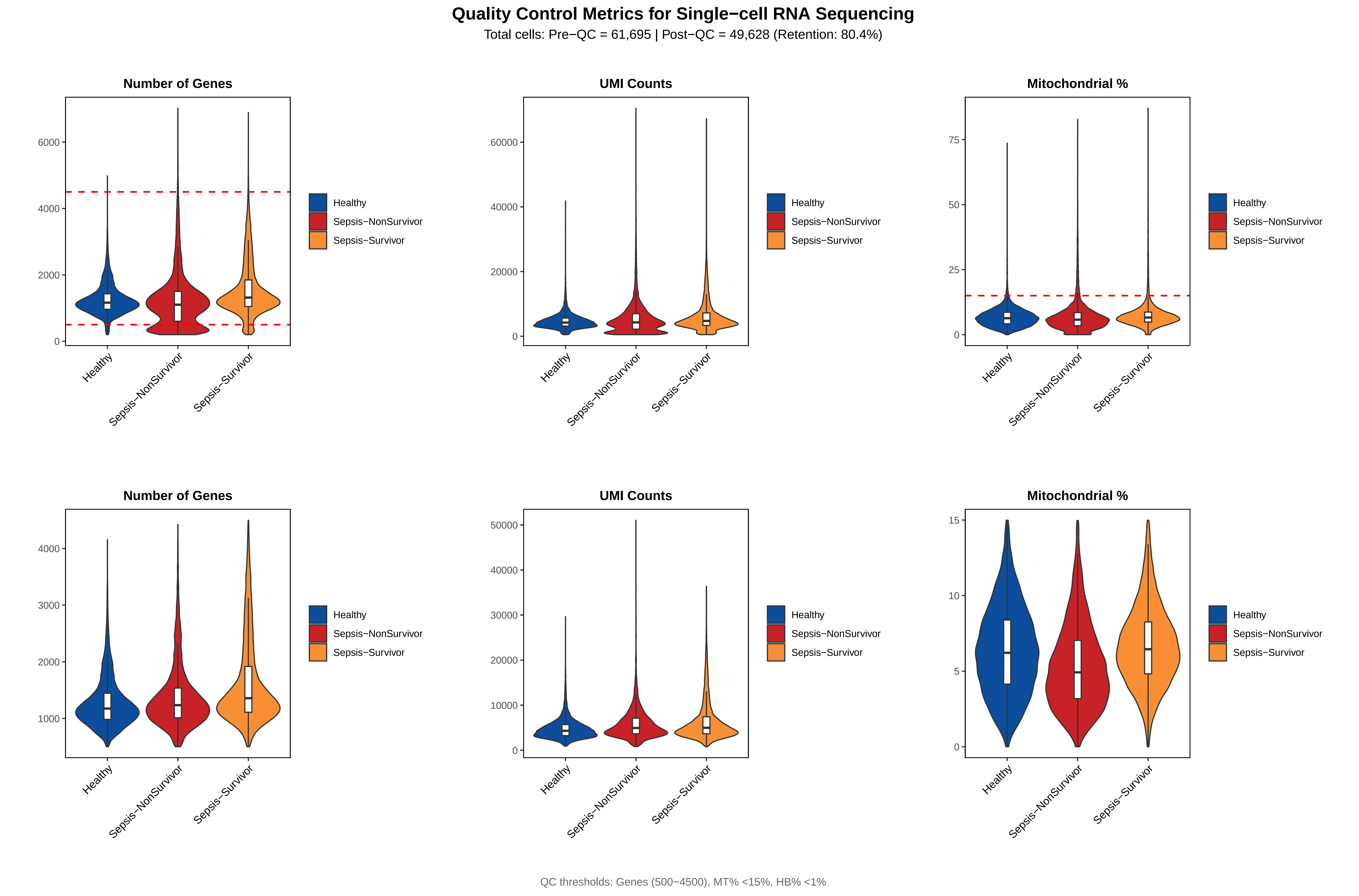
Quality Control Metrics for Single−cell RNA Sequencing
Total cells: Pre−QC = 61,695 | Post−QC = 49,628 (Retention: 80.4%)
Number of Genes
UMI Counts
Mitochondrial %
Healthy
Sepsis−NonSurvivor
Sepsis−Survivor
QC thresholds: Genes (500−4500), MT% <15%, HB% <1%

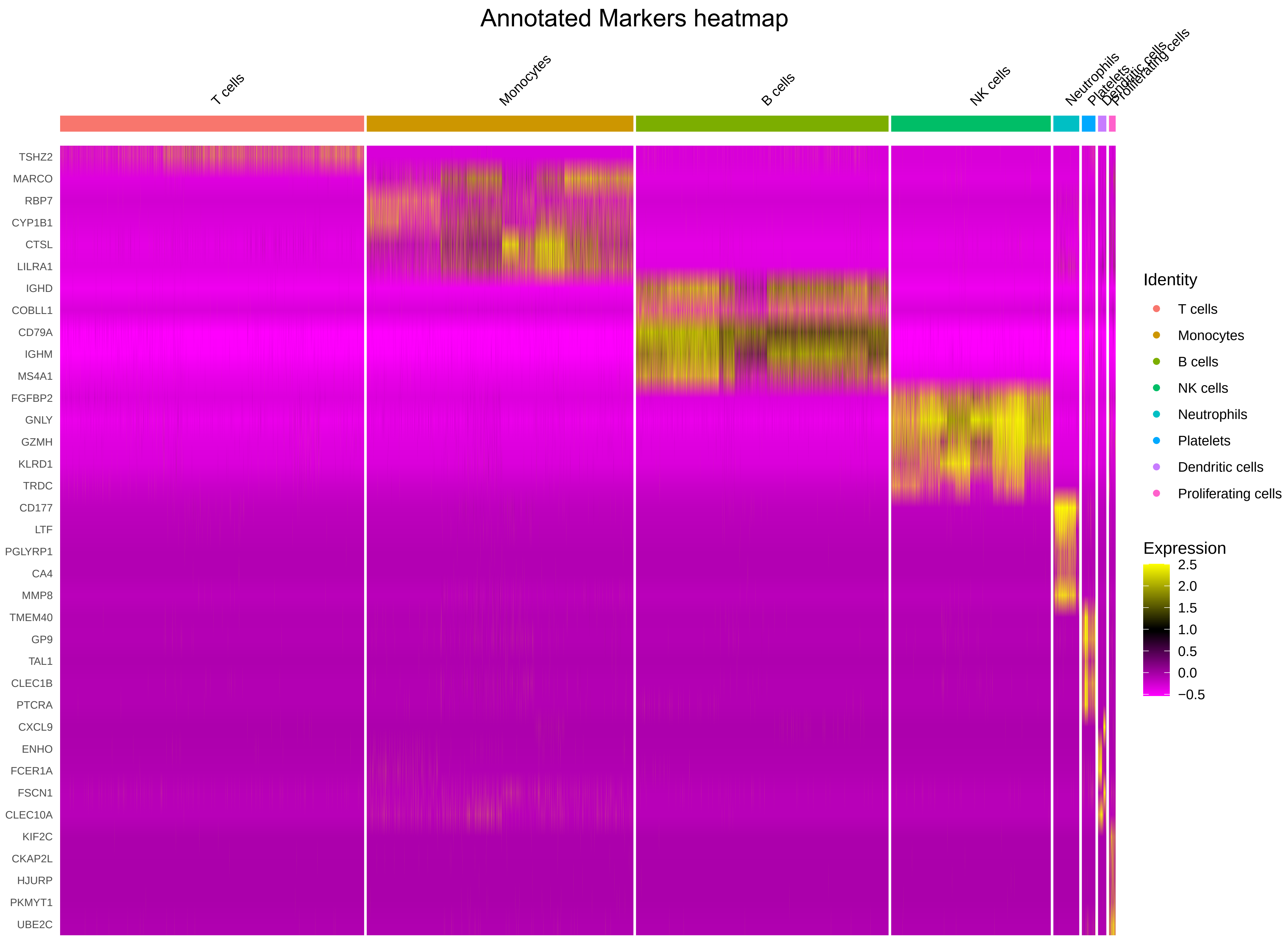
Annotated Markers heatmap
T cells
Monocytes
B cells
NK cells
Neutrophils
Platelets
Dendritic cells
Proliferating cells
TSHZ2
MARCO
RBP7
CYP1B1
CTSL
LILRA1
IGHD
COBLL1
CD79A
IGHM
MS4A1
FGFBP2
GNLY
GZMH
KLRD1
TRDC
CD177
LTF
PGLYRP1
CA4
MMP8
TMEM40
GP9
TAL1
CLEC1B
PTCRA
CXCL9
ENHO
FCER1A
FSCN1
CLEC10A
KIF2C
CKAP2L
HJURP
PKMYT1
UBE2C
Identity
T cells
Monocytes
B cells
NK cells
Neutrophils
Platelets
Dendritic cells
Proliferating cells
Expression
2.5
2.0
1.5
1.0
0.5
0.0
−0.5

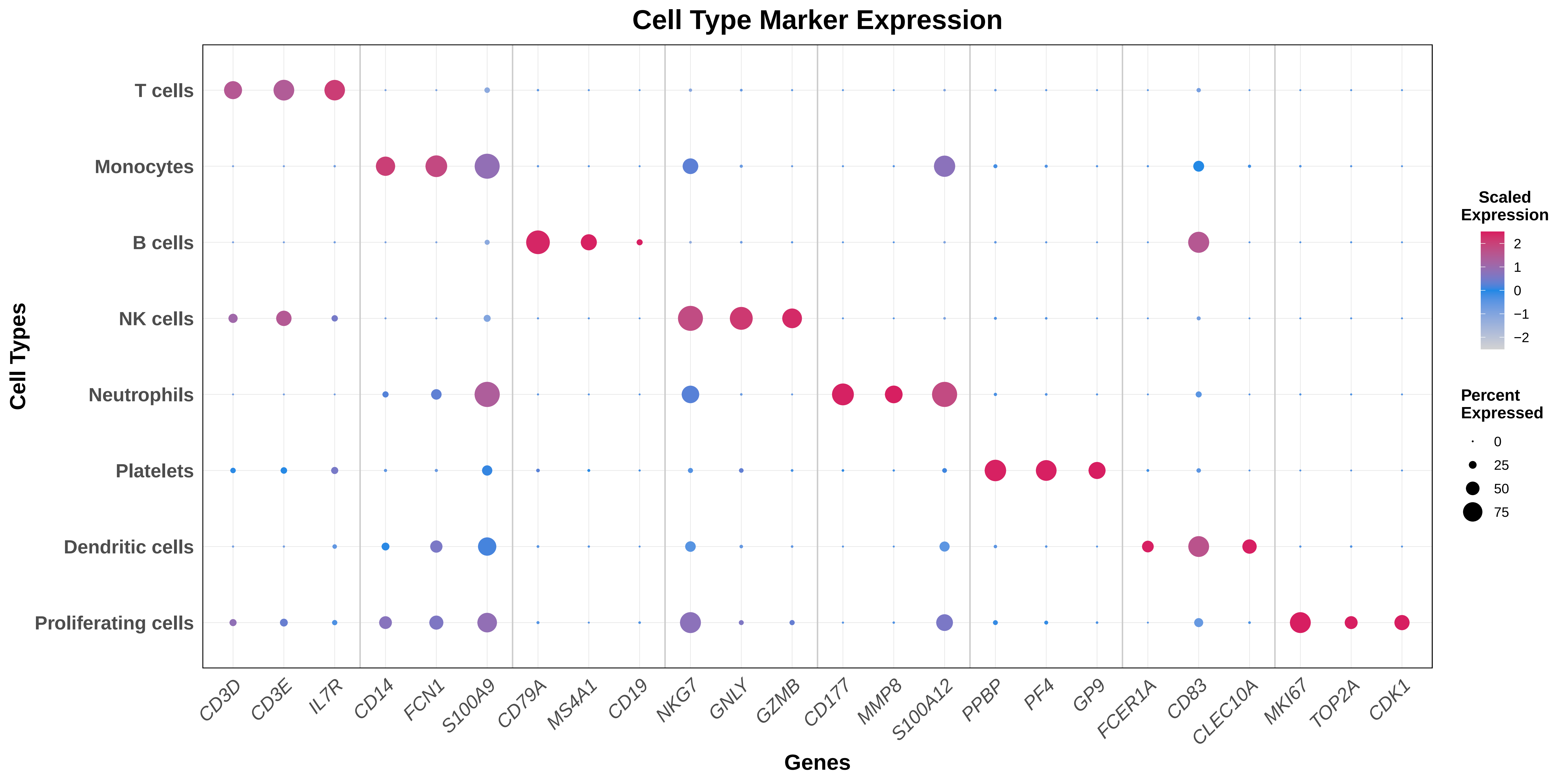

Cell Type Marker Expression
Cell Types
T cells
Monocytes
B cells
NK cells
Neutrophils
Platelets
Dendritic cells
Proliferating cells
Genes
CD3D
CD3E
IL7R
CD14
FCN1
S100A9
CD79A
MS4A1
CD19
NKG7
GNLY
GZMB
CD177
MMP8
S100A12
PPBP
PF4
GP9
FCER1A
CD83
CLEC10A
MKI67
TOP2A
CDK1
Scaled Expression
2
1
0
−1
−2
Percent Expressed
0
25
50
75

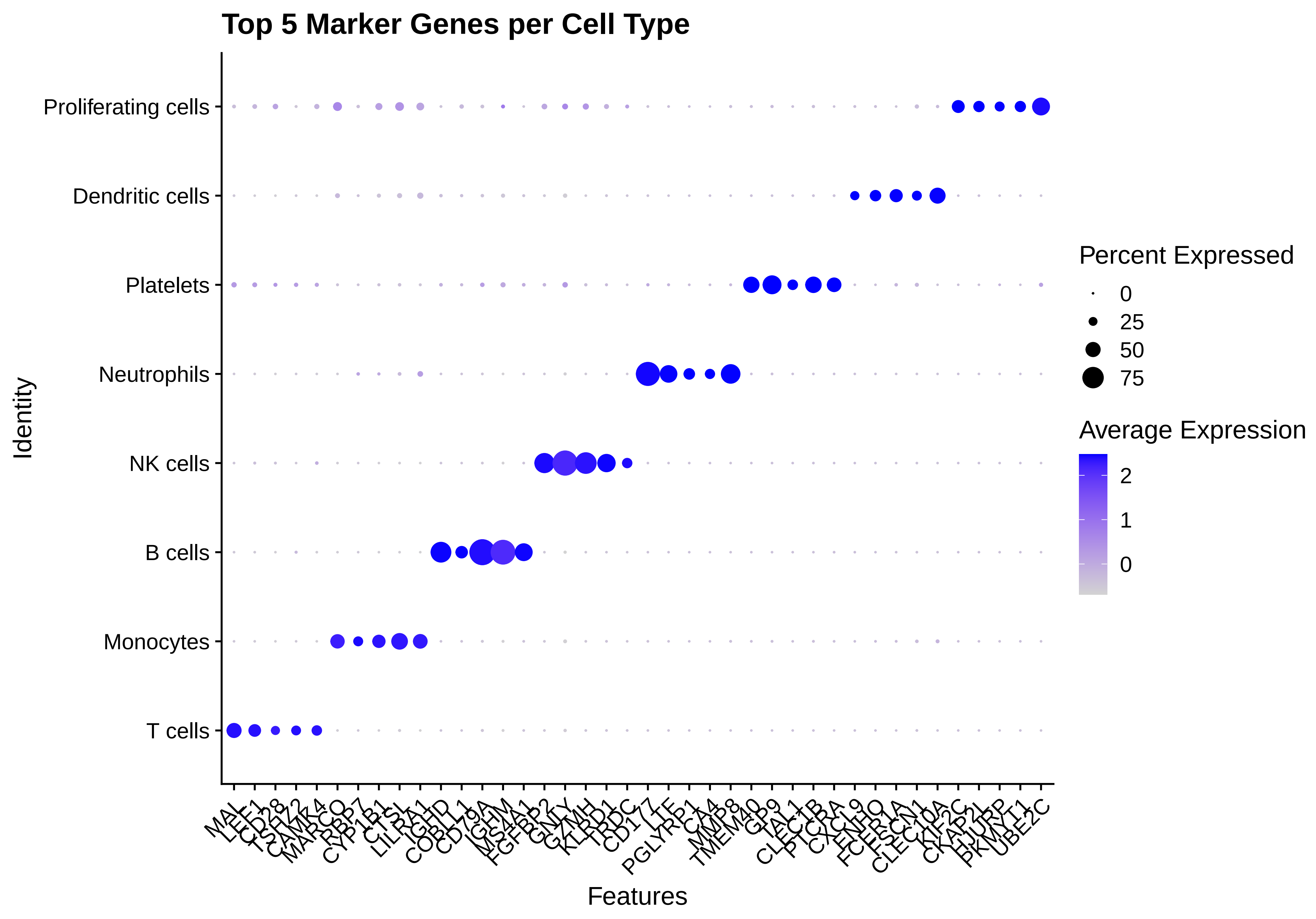

Top 5 Marker Genes per Cell Type
Identity
Proliferating cells
Dendritic cells
Platelets
Neutrophils
NK cells
B cells
Monocytes
T cells
MAL
LEF1
CD28
TSHZ2
CAMK4
MARCO
RBP7
CYP1B1
CTSL
LILRA1
IGHD
COBLL1
CD79A
IGHM
MS4A1
FGFBP2
GNLY
GZMH
KLRD1
TRDC
CD177
LTF
PGLYRP1
CA4
MMP8
TMEM40
GP9
TAL1
CLEC1B
PTCRA
CXCL9
ENHO
FCER1A
FSCN1
CLEC10A
KIF2C
CKAP2L
HJURP
PKMYT1
UBE2C
Features
Percent Expressed
0
25
50
75
Average Expression
2
1
0
